# Sirtuin-dependent metabolic and epigenetic regulation of macrophages during tuberculosis

**DOI:** 10.3389/fimmu.2023.1121495

**Published:** 2023-03-13

**Authors:** Kangling Zhang, Mark L. Sowers, Ellie I. Cherryhomes, Vipul K. Singh, Abhishek Mishra, Blanca I. Restrepo, Arshad Khan, Chinnaswamy Jagannath

**Affiliations:** ^1^ Department of Pharmacology and Toxicology, University of Texas Medical Branch, Galveston, TX, United States; ^2^ Department of Pathology and Genomic Medicine, Houston Methodist Research Institute, Weill-Cornell Medicine, Houston, TX, United States; ^3^ University of Texas Health Houston, School of Public Health, Brownsville, TX, United States

**Keywords:** human macrophages, autophagy, glycolysis, metabolism, histone modifications, SIRTUIN

## Abstract

Macrophages are the preeminent phagocytic cells which control multiple infections. Tuberculosis a leading cause of death in mankind and the causative organism *Mycobacterium tuberculosis* (MTB) infects and persists in macrophages. Macrophages use reactive oxygen and nitrogen species (ROS/RNS) and autophagy to kill and degrade microbes including MTB. Glucose metabolism regulates the macrophage-mediated antimicrobial mechanisms. Whereas glucose is essential for the growth of cells in immune cells, glucose metabolism and its downsteam metabolic pathways generate key mediators which are essential co-substrates for post-translational modifications of histone proteins, which in turn, epigenetically regulate gene expression. Herein, we describe the role of sirtuins which are NAD^+^-dependent histone histone/protein deacetylases during the epigenetic regulation of autophagy, the production of ROS/RNS, acetyl-CoA, NAD^+^, and S-adenosine methionine (SAM), and illustrate the cross-talk between immunometabolism and epigenetics on macrophage activation. We highlight sirtuins as emerging therapeutic targets for modifying immunometabolism to alter macrophage phenotype and antimicrobial function.

## Introduction

Macrophages are the preeminent phagocytic cells which respond to pathogen invasion using a variety of anti-microbial mechanisms. Circulating monocytes originating in bone marrow become macrophages (MΦs) at tissue sites of infection after getting exposed to cytokines and microbial stimuli. During tuberculosis, the causative organism *Mycobacterium tuberculosis* (MTB) infects and grows in naive MΦs. That tuberculosis continues to kill more than a million people each year indicates that MTB has evasion mechanisms to survive and grow in MΦs. Indeed, MTB evades antimicrobial mechanisms of MΦs using multiple strategies including epigenetic modifications (1, 2). For example, MTB encodes for dozens of methyltransferases of which, products from *Rv1988* and *Rv2966c* methylate DNA (2, 3); DNA hypermethylation of MΦs was reported to decrease immunity in TB patients (4). MTB derived *Rv3423.1* acetylates histones affecting gene expression, whereas Enhanced intracellular survival (Eis) protein acetylates histone H3 at K9 and K14 and increases IL-10 (5). Together, these observations suggest that ‘acetylation and methylation’ are important for controlling antimicrobial mechanisms within MΦs during intracellular infections like tuberculosis.

Intriguingly, T cell derived cytokines like IFN-γ drive naïve MΦs into an M1-MΦ phenotype whereas, IL-4, IL-10 and IL-13 differentiate them into M2-MΦs. We recently reported that MTB infected human M1- and M2-MΦs show unique transcriptional responses and M1-MΦs were able to inhibit the growth of MTB using a nitric oxide- and autophagy-dependent mechanism, whereas M2-MΦs were permissive for growth (6). During these studies, we noted that M1- and M2-MΦs expressed differing levels of sirtuin (SIRT) histone/protein deacetylases and significantly, SIRT2 blockade increased the ability of MΦs to kill MTB suggesting a pivotal role.

Recent studies demonstrate that the activity of MΦ derived histone acetyltransferases is regulated by their co-substrate, acetyl-CoA (ac-CoA), whereas the activity of sirtuin proteins which are nicotinamide adenine dinucleotide (NAD^+^)-dependent histone deacetylases, is dependent on NAD^+^. It is also known that the activity of histone methyltransferases and DNA methyltransferases is regulated by their specific co-substrate, s-adenosylmethionine (SAM) (7). Therefore, chromatin-modifying enzymes can sense the metabolic status and translate this information into gene expression. In MФs, this would determine their polarization state as either pro-inflammatory IFN-γ/LPS inducible M1-MΦs or alternatively activated and anti-inflammatory, IL-4/IL-10 and IL-13 driven M2-MΦs. Interestingly, Glucose metabolism differs between M1- and M2-MΦs and glycolysis and its associated pentose-phosphate- pathway (PPP), serine biosynthesis, and one-carbon metabolism are major sources for the co-substrates for methylation and acetylation. In M1-MΦs, glucose uptake is elevated by up-regulated glucose transporter GLUT1, followed by up-regulated glycolysis (8, 9). High glucose intake and metabolism is essential for phagocytosis, production of reactive-oxygen-species (ROS) and reactive-nitrogen-species (RNS), and secretion of pro-inflammatory cytokines (10).

Emerging evidence also links glycolysis to epigenetics. Locasale’s and Schultz’s groups have demonstrated that histone acetylation is enhanced by glucose flux in a variety of cell types (11, 12). Acetylation is strongly associated with ac-CoA levels but inversely correlated with the ratio of ac-CoA to free CoA (11). Inhibition of glycolysis results in the reduced production of ac-CoA and reduction of histone acetylation leading to differentiation of embryonic stem cells (13). In bacteria, two-thirds of glycolytic and TCA cycle enzymes are acetylated, with acetylation inhibiting their catalytic activity and promoting degradation (14). Glycolysis also regulates histone deacetylation because NAD^+^-dependent srtuin proteins regulate the expression of glycolytic enzymes and the ratio of NAD^+^/NADH is controlled by the glycolytic flux, and vice versa (15–18). In addition to acetylation, glycolysis also indirectly affects methylation through serine biosynthesis that utilizes 3-phospho-glycerate (3-P-G) as the starting material (19). Through one-carbon metabolism, serine is used for the *de novo* synthesis of methionine and SAM which is the co-substrate of methyltransferases (19, 20).

In this review, we summarize the recent research on the regulation of glucose metabolism and its associated metabolism by sirtuin proteins and their co-substrate NAD^+^ and their impact on epigenetic regulation of MΦ activation, polarization, and autophagy activity. We also discuss the NAD^+^-dependent sirtuin histone deacetylases as emerging drug targets for the treatment of infectious diseases, specifically for tuberculosis. Since we wish to focus on metabolism-derived co-substrates of histone acetylation/methylation enzymes and the NAD^+^-dependent histone deacetylase-sirtuin proteins, epigenetic regulation of autophagy by other histone modification enzymes or modification states is beyond the scope of this review and are covered elsewhere (21, 22).

## Glucose metabolism and immune responses

**Figure 1:**
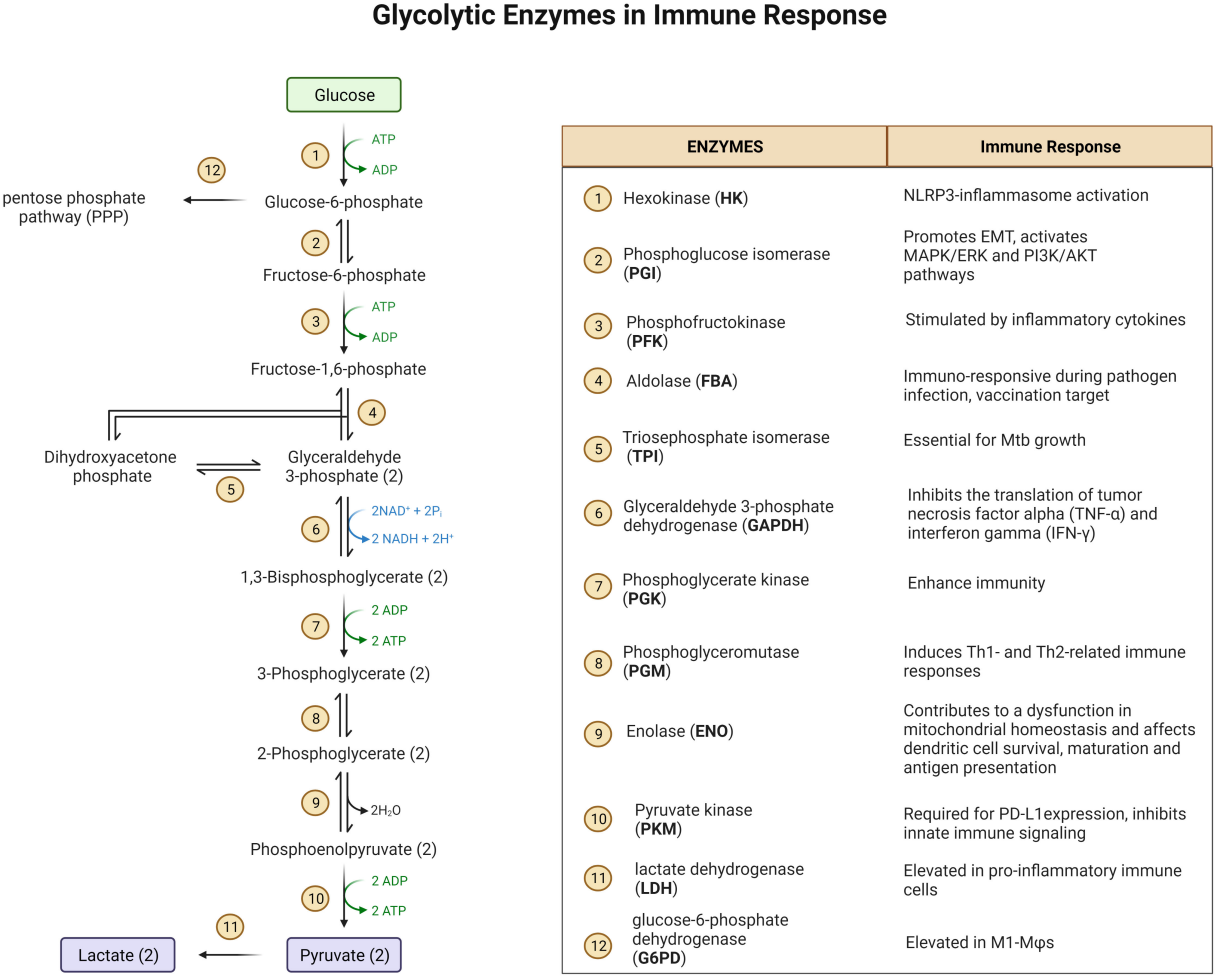
A diagram of glycolysis and glycolic enzymes involved in the regulation of immune responses.

Glucose metabolism exerts a strong impact on immune cell function (**Figure 1**) (23). For example, hexokinase (HK) binds to bacterium-produced N-acetylglucosamine and causes its deactivation as well as its dissociation from mitochondrial voltage-dependent anion channels (VDACs), which in turn, leads to NOD-Like Receptor family Pyrin domain containing 3 (NLRP3) inflammasome activation in MΦs (24, 25). Phosphoglucose isomerase (PGI) is identical to the protein known as Autocrine Motility Factor (AMF) which is upregulated in cancer cells together with other glycolysis enzymes and thought to play a key role in cancer metastasis by activating Epithelial-Mesenchymal Transition (EMT) and the MAPK/ERK or PI3K/AKT pathways (26, 27). These pathways are also upregulated in glucose deprived MΦs (28–30). Fructose-bisphosphate aldolase (FBA) is immuno-responsive during pathogen infection and is a potential vaccination target (31). Triosephosphate isomerase (TPI) catalyzes the interconversion of dihydroxyacetone phosphate (DHAP) and glyceraldehyde-3-phosphate (G3P). TPI has been predicted to be essential for growth of MTB (32). Phosphoglycerate kinase (PGK) enhances the immunity to *Streptococcus agalactiae* in tilapia (33). Immunization of phosphoglyceromutase (PGM) induces Th1- and Th2-related immune responses in mice infected with Brucella (34). Deficiency of enolase (ENO1) causes the reduction of pyruvate which then contributes to a dysfunction in mitochondrial homeostasis and affects dendritic cell survival, maturation and antigen presentation (35). Pyruvate kinase 2 (PKM2) is required for the expression of PD-L1 in immune cells and tumors. Loss of PKM2 impairs endothelial cell proliferation and migration and triggers innate immune signaling (36). Glyceraldehyde-3-phosphate dehydrogenase (GAPDH) binds to 3’-UTR of inflammatory mRNAs and inhibits the translation of tumor necrosis factor alpha (TNF-α) and interferon gamma (IFN-γ) (37). PDK2/4 serves as a metabolic checkpoint for polarization of macrophages into the pro-inflammatory M1 phenotype (38). Though not generally characterized as a glycolytic enzyme involved in the 10 steps of glycolysis, lactate dehydrogenase (LDH) is elevated in pro-inflammatory immune cells to produce surplus lactate. Another glycolysis-related enzyme is glucose-6-phosphate dehydrogenase (G6PD) which acts at the first and the rate-limiting step of the pentose phosphate pathway (PPP). G6PD level is elevated in M1-MΦs and cells deficient in G6PD have a reduced ability to induce the innate immune response, thereby increasing host susceptibility to infection with pathogens (39). In addition to glycolytic enzymes, the glycolytic intermediates also play a significant role in the activation of the immune system. Pyruvate is reduced by LDH to form lactate that regulates immune response in macrophages and dendritic cells (40). Importantly, phosphoenolpyruvate (PEP), the precursor of pyruvate, is an immune signaling molecule; it promotes pro-inflammatory functions and activates T cells by regulating Ca^2+^-transportation and translocation of nuclear factor of activated T cells (NFAT) (41). Other metabolic enzymes and their products associated with glucose and immunometabolism have been reviewed elsewhere. These pathways include PPP (42), TCA cycle (43, 44), serine biosynthesis and one-carbon metabolism (45, 46), glutamine metabolism (47), and arginine metabolism (48).

## Glucose metabolism, reactive oxygen (ROS) and reactive nitrogen species (RNS) production in macrophages, and their action on bactericidal function

**Figure 2:**
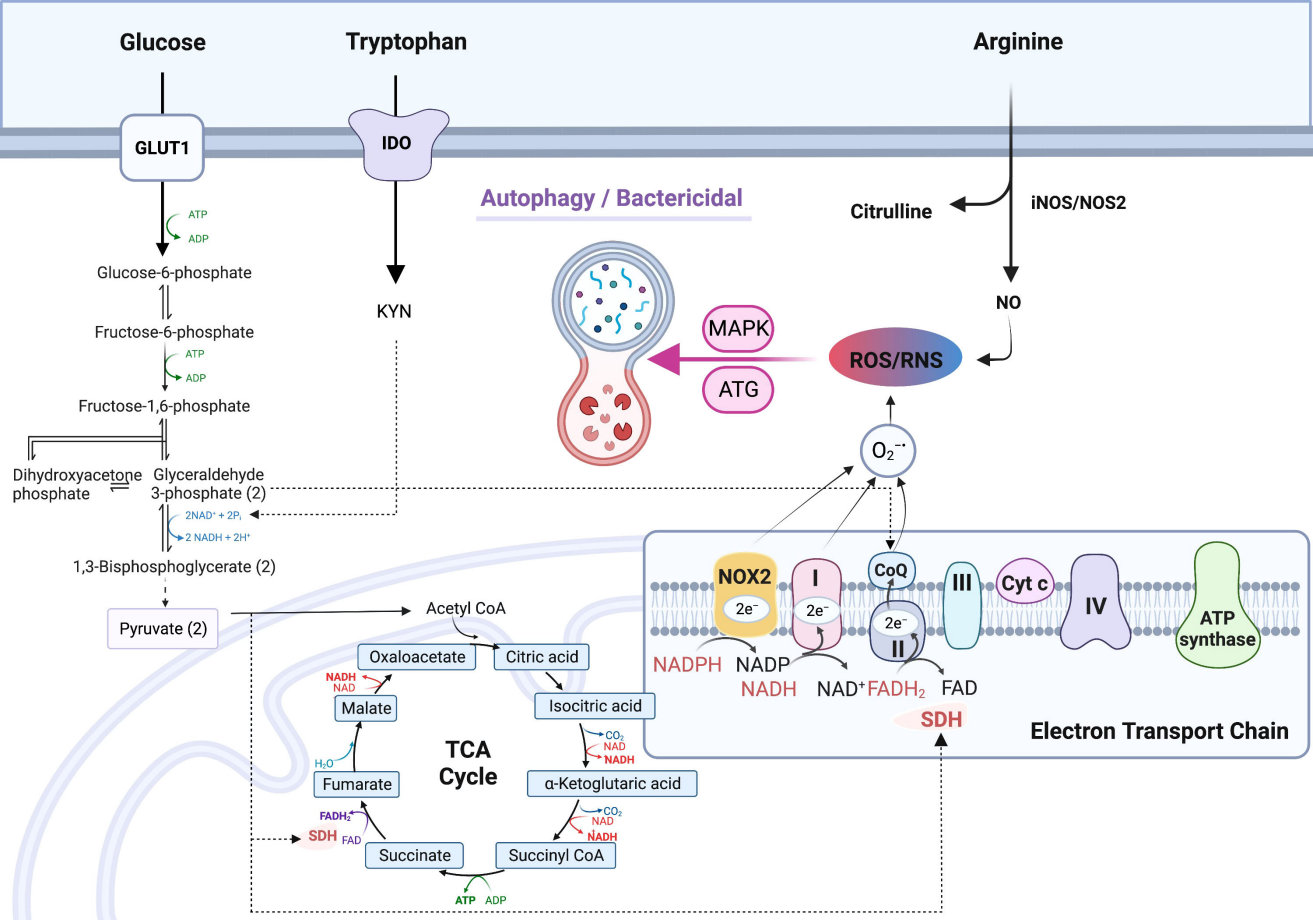
Up-regulated production of reactive oxygen species (ROS) and reactive nitrogen species (RNS) in M1- macrophages. Increased production of NADH from reduction of NAD^+^ *via* glycolysis fuels the electron transport chain (ETC) complex 1 to generate O_2_^.-^; NAD^+^ is continuously replenished by upregulated NAD^+^ *de novo* synthesis from tryptophan metabolism. Increased production of NADPH *via* the pentose-phosphate-pathway (PPP), which is also up-regulated in M1- type macrophages (MΦs), fuels the ETC to produce O_2_^.-^. O_2_^.-^ is also generated *via* succinate dehydrogenase (SDH) which is coupled with FADH2/FAD redox reaction in complex II of ETC. In M1-MΦs, RNS is derived from nitric oxide (NO) which is produced by arginine metabolism through iNOS/NOS2. Mitochondrial ROS/RNS regulates phagocytosis, bacterial killing, and polarization towards M1-MΦs *via* ATG and MAPK activation. *Additional symbols:* 1,3-BPG, 1,3-bisphosphoglyceric acid; α-KG, alpha-ketoglutarate; ATG, Autophagy regulating gene; CIT, citrate; DHAP, dihydroxyacetone phosphate; F-1, 6-P, Fructose-1, 6-biphosphate; F-6-P, Fructose-6-phosphate; FUM, fumarate; G-3-P, glycerol-3-phosphate; G-6-P, glucose-6-phosphate; GABA, γ-aminobutyrate; GA, glutaminase; GABA-T, GABA transferase; GAD, glutamate decarboxylase; GDH, Glutamate dehydrogenase; Gln, glutamine; Glu, glutamate; GLUT1, glucose transporter protein type 1; iCIT, isocitrate; IDO, Indoleamine-pyrrole 2,3-dioxygenase; Kyn, kynurenine; Lac, lactate; MAL, malate; OAA, oxaloacetic acid Pyr, pyruvate; SSA, succinate semialdehyde; SSADH, succinate semialdehyde dehydrogenase; SUC-CoA, succinyl-CoA; SUC, succinate.

Reduction-oxidation (redox) reactions occur in various metabolic processes including glycolysis, TCA cycle, but predominantly in the electron-transport chain (ETC) of mitochondria, which is essential for the generation of energy (ATP) for living cells. Oxidants, typically reactive oxygen species (ROS), are produced as the byproducts of redox reactions in ETC (49, 50). The major cellular redox reactions are conversions between NAD^+^ and NADH, NADP^+^ and NADPH, and FAD and FADH2. NAD^+^ is reduced/converted into NADH during glycolysis (two molecules) and in TCA cycle (three molecules) (51). NADH is re-oxidized to NAD^+^ by either lactate dehydrogenation (LDH) which catalyze the conversion of pyruvate into lactate, or by the ETC complex I through which, one proton and two electrons are released and ROS (O_2_^.-^) is formed when the electrons are added to O_2_ (51, 52). Paralleling the glycolysis initiating at glucose-6-phosphate, the pentose-phosphate-pathway (PPP) generates NADPH (from NADP^+^) from pentose as well as ribose 5-phosphate, a precursor for the synthesis of nucleotides. Similar to NAD^+^/NADH, NADPH is oxidized by NADPH oxidase (NOX) to be converted back to NADP^+^ and ROS is formed when the released electrons are added to O_2_ (53). NOX is a membrane-bound flavocytochrome, containing two molecules of heme and one molecule of flavin adenine dinucleotide (FAD) with a spectroscopic absorbance^max^ of 558 nm. For this reason, NOX is also referred to as flavocytochrome b558 which contains p22^phox^ (α-subunit, the production of the CYBA gene) and NOX2/gp91^phox^ (β-subunit, CYBB gene) (54). NOX is found in functional phagocytes including neutrophils, eosinophils, monocytes, dendritic cells, and macrophages (55). The third pair of redox reaction is FAD and FADH2, which are bound to succinate dehydrogenase complex (SDH). The substrate of SDH is succinate, an intermediate of TCA cycle, which is synthesized directly from succinyl-CoA. Succinate synthesis is enhanced in M1-MΦs due to the inhibition of TCA cycle (9). In addition, succinate can also be synthesized *via* glutamine-dependent anaplerosis or the γ-aminobutyric acid (GABA) shunt, which promotes and maintains polarization of M1-MΦs (56). SDH is a part of ETC Complex II, and mediates oxidation of succinate into fumarate. This reaction is coupled with the reduction of ubiquinone (UQ) to ubiquinol (UQH2) coupling with the oxidation of FADH2 to FAD. When high amounts of succinate are oxidized to fumarate under low oxidative phosphorylation conditions, electron flux moves in the opposite direction of ETC, from complex II toward complex I, leading to reverse electron transport (RET) and generating ROS (9, 57, 58) (**Figure 2**). The production of mitochondrial ROS is also mediated by immunoresponsive gene 1 (IRG1), which utilizes β-oxidation of fatty acids to generate ROS and improved activity of ETC increases ROS production in phagosomes thereby augmenting bactericidal activity (59). On the other hand, IRG1 is also called Aconitate Decarboxylase 1 (ACOD1), an important enzyme in the TCA cycle, which converts aconitate to itaconic acid that has a canonical antibacterial role through isocitrate lyase inhibition (60, 61). IRG1 is specifically up-regulated in LPS induced pro-inflammatory murine M1-MΦs (62, 63). ROS are essential for macrophages to fight against invasive pathogens through the M1-MΦ-dependent innate immune defense system, but they also play a critical role in signal transduction, differentiation, and gene expression (64, 65). In addition to ROS, cells generate oxidants through reactive nitrogen species (RNS). RNS are produced from the reaction of nitric oxide (•NO) with superoxide (O_2_^•−^) to form highly reactive peroxynitrite (ONOO^−^) (66) (**Figure 2**). NO is synthesized from arginine by NO synthase (NOS2/iNOS) (67, 68). Macrophages produce both ROS and RNS in response to phagocytosis and are required for killing of pathogens (69). The antimicrobial function of macrophages mainly depends on NOS2 and NOX2 genes which are upregulated in both murine and human M1-MΦs to generate abundant ROS and RNS (70–74). Therefore, M1-MΦs exhibit a high bactericidal function to defend against many intracellular pathogens including MTB (75). It has been noted that M1-MΦs have lower acidification rate and reduced proton pumping activity and thereby increased proton moving force compared to M2-MΦs; this facilitates M1-MΦs to generate ROS and efficiently control pathogens (76). Interestingly, NO also enhances the accumulation of itaconic acid in inflammatory cells increasing anti-bacterial activity (77); consequently, gene disruption of IRG1 reduces itaconic acid increasing the susceptibility to MTB infection and lung immunopathology (78). Paradoxically, for some pathogens, excess ROS can hijack host immune system and become favorable to pathogen survival (79). The mechanisms of ROS dependent hijack are not clear but inhibition of ETC complex I and regulation of TCA intermediates by NO may provide a plausible explanation (77, 80).

## Metabolic profiles of mouse and human M1- versus M2-MΦs during tuberculosis infection

Metabolic gene expression profiling has revealed a biphasic metabolic behavior of MTB infection using an animal model (81). In the early phase post infection (up to 8 hr), the innate immune system is activated to generate proinflammatory cytokines including interleukin-1β (IL-1β), IL-6, IL-12, and TNF-α, predominantly in the M1-MΦs. In this early phase, glucose uptake aided by upregulated GLUT1 is accelerated and the genes of glycolysis are activated to increase the production of ATP and glycolytic intermediates and increase the consumption of NAD^+^. Concurrently, oxidative metabolism is down regulated indicated by a decrease in key enzymes of the TCA cycle and mitochondrial ETC complexes in mice exposed to MTB (82–84). However, as the infection progresses to 24 and 48 hr, post-infection, the M1- metabolic state of macrophages is reversed and an increase in TCA cycle and oxidative phosphorylation with dampened glycolysis are observed suggesting a switch towards M2-MΦs (9, 81, 85). These data are consistent with increased glycolysis and reduced TCA proteins in human M1-MΦs and switch towards M2-MΦs observed using proteomics analysis in our lab (86). Whereas most proteins of the ETC complexes II-IV were down-regulated, majority of proteins in complex I were up-regulated in human M1-MΦs (86).

Although many metabolic profiling studies have been done using mouse macrophages, recent studies are focusing on human macrophages (86–92). For example, mice are more susceptible to tuberculosis whereas nearly 90% of humans exposed to tuberculosis develop latent infection indicating a better control by their macrophages. In this direction, Gleeson, et al. identified that lactate derived from glycolysis-generated pyruvate, is increased in M1-MΦs when activity of TCA cycle is down-regulated, suggesting it as a key player during metabolic remodeling in MTB-infected human macrophages (93). Treatment of resting human macrophages with exogenous lactate caused a decrease in extracellular acidification rate while an increase of oxygen consumption rate (analogous to oxidative phosphorylation), resulted in an increased capacity to kill MTB possibly through autophagy (94). The same study also found that tuberculosis antimicrobial drugs, such as clofazimine, reshaped the immunometabolic profiles of MTB infected human macrophages towards oxidative phosphorylation similar to the effects of lactate (95).

On the other hand, Cumming, et al. found that in MTB-infected human monocyte-derived macrophages (nondifferentiated/resting state) both glycolysis and oxidative phosphorylation were suppressed leading to a state of metabolic quiescence resulting in a decrease of ATP production in mitochondria and a switch from dependency on glucose to fatty acids (88). This study suggested that MTB promoted polarization of macrophages towards M2-MΦs. We suggest that this discrepancy could arise when the starting monocyte-macrophage populations are different. Nonetheless, there seems to be a consensus that MTB infected human macrophages undergo a transition from M1-MΦs during early phase of infection to M2-MΦs during late phase similar to the mouse data (81).

Interestingly, pharmaceutical modulation with histone deacetylase inhibitor, suberanilohydroxamic acid (SAHA) promoted the glycolysis rate of human macrophages with increased production of pro-inflammatory cytokine IL-1β (a marker of M1-MΦs) and decreased production of anti-inflammatory cytokine IL-10 (a marker of M2-MΦs) during the early stage of MTB infection associated with enhanced T helper cell responses *ex vivo* (87). In this direction, we recently reported RNA-seq based transcriptomic data supporting metabolic profiling; genes of glycolysis, TCA cycle, and ETC complexes were all up-regulated that MTB infected in M1-MΦs at 24 hr post infection (6). We further demonstrated that human M1-MΦs expressed unique innate immune response genes to defend against tuberculosis through increased production of NO, accelerated autophagy- dependent killing of MTB and increased antigen presentation to T cells through an *ATG-RAB7*-cathepsin pathway (6). Taken together, these data indicate that MTB infection promotes naïve macrophage polarization progressively from M1-MΦ to M2-MΦ phenotype. The biphasic metabolic switch observed using *ex-vivo* MTB-infected human macrophages is similar to that of mouse macrophages infected with MTB. However, mice still develop progressive tuberculosis after aerosol infection with MTB unlike humans suggesting that differences in metabolic regulation of M1- *vs*. M2-MΦs may exist. For example, we found that sirtuins were differentially expressed by MTB infected M1 and M2-MΦs unlike similarly infected in mouse MΦs (96). The metabolic basis for the differences in the antimicrobial function of M1 *vs*. M2-MΦs is discussed below.

## Glucose has a profound impact on immunometabolism and autophagy in human MΦs

**Figure 3:**
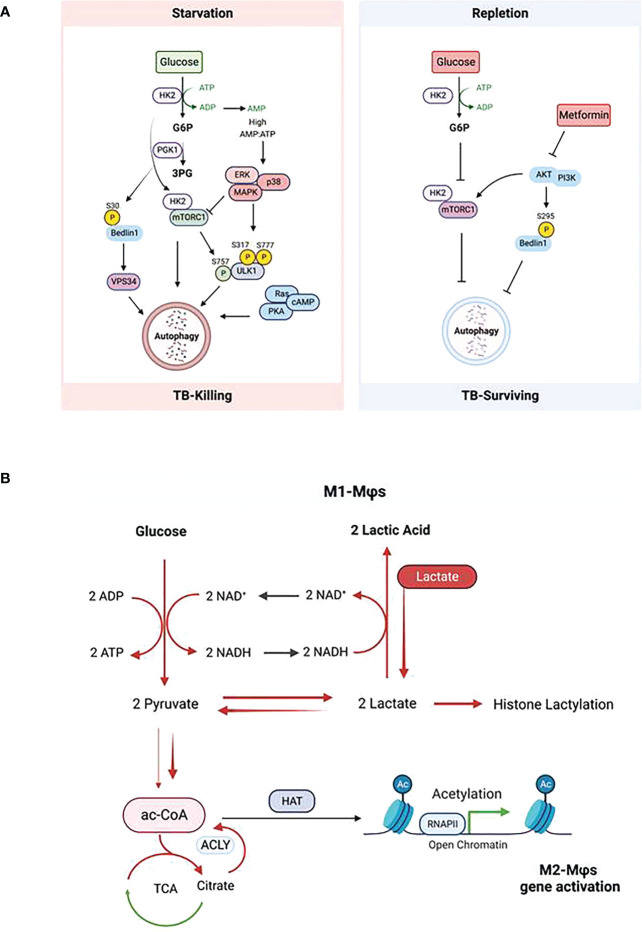
Impact of glucose metabolism on autophagy in macrophages during tuberculosis. **(A)** Regulation of autophagy by glucose homeostasis (*Left*: glucose starvation; *Right*: glucose repletion) dependent metabolic sensor kinases. Description of the scheme is referred to the text. **(B)** Glycolysis-promoted histone lactylation and acetylation during macrophage polarization. Upregulation of glycolysis in M1-Mϕs increases lactate production from pyruvate; however, excess of lactate provided exogenously pushes the equilibrium of the conversion between pyruvate and lactate further to the synthesis of citrate from pyruvate and the former is broken up to acetyl -CoA by ACLY; this results in the elevation of both global histone acetylation and acetylation of chromatins associated with the promoters of genes which promote polarization towards M2-Mϕs.

Glucose metabolism and glycolysis are key players in inflammatory response (10). In mouse M1-MΦs exposed to pathogens, both glycolysis and GLUT1 expression are upregulated in the early phase of infection to facilitate rapid glucose uptake and consumption, resulting in eventual depletion of glucose, increased acidification of the microenvironment, both of which can be detrimental to proliferating pathogens (8, 9, 97). In addition to ROS/RNS discussed above, another bactericidal mechanism of macrophages is autophagy which is regulated by nutritional and metabolic states (98). Autophagy is generally induced by decreased availability of glucose or other nutrients such as amino acids (**Figure 3A**). In contrast, it can be stimulated by metabolites such as fatty acids and ammonia. Under nutrition-restricted conditions, glucose, acetyl-CoA, and amino acids are depleted, and NAD^+^ accumulates leading to an increase in the NAD^+^/NADH ratio (51) which in turn, regulates autophagy (99, 100). Several metabolic-sensor kinases also regulate this process (**Figure 3A**).

The target of rapamycin complex 1 (mTORC1) of the mTOR complex is a positive regulator of glycolysis and is activated in M1-MΦs. Whereas mTORC1 inhibits autophagy, inhibition of mTORC2 activates the process. Roberts et al. demonstrated that during glucose-limiting conditions, HK2 binds and inhibits mTORC1 thereby activating autophagy, whereas in glucose-repletion condition, glucose-6-phosphate (G6P) inhibits the binding of HK2 to mTORC1 to suppress autophagy (101, 102). Therefore, HK2 and G6P are pharmaceutical targets to induce autophagy in glucose-rich condition. Indeed, Metformin, a biguanide antidiabetic drug, lowers G6P in hepatocytes by activation of glucose phosphorylation, which is downstream of glycolysis and triggers autophagy (103, 104). This means that during early phase of infection of macrophages, high glucose intake produces excess ATP that activates ROS-dependent oxidative stress response and thereby up-regulated pro-inflammatory cytokines but this process also reduces autophagy without pharmacological intervention (102, 105). However, during the later phase of infection of macrophages, glucose and other nutrients are depleted, resulting in activation of autophagy and ROS level.

The second class of kinases is the AMP-activated kinases (AMPKs) which activate autophagy. Under glucose starvation, AMPK promotes autophagy by directly activating ULK1 through phosphorylation of Ser317 and Ser777 (106, 107), which can be prevented by mTORC1 that phosphorylates Ulk1 at Ser757 (106, 107). Glycolysis provides most of ATP in M1-MФs which is hydrolyzed into ADP and further into AMP, generating energy needed by cells. During glucose starvation, the AMP/ATP ratio increases leading to the activation of AMPK (108). Activation of AMPK inhibits mTOR resulting in an increase of autophagy (109, 110). Seemingly redundant to mTORC1, the RAS/cAMP-dependent protein kinase A (PKA) signaling pathway also regulates the induction of autophagy in yeast and mammals (111, 112). In addition to mTORC1 and PKA, Akt in the PI3K/Akt signaling pathway also regulates autophagy. Akt inhibits autophagy through phosphorylating the C-terminal Ser279 of Beclin-1 in the core autophagy machinery independent of mTORC1 (113, 114). Interestingly, during glutamine deprivation or hypoxia, a glycolytic enzyme – phosphoglycerate (PGK1), also directly phosphorylates the N-terminal Ser30 of Beclin-1 leading to enhanced VPS34 activity and subsequent autophagy (115). Of note, phosphorylation of Beclin-1 at N-terminus or C-terminus has different effects on autophagy; phosphorylation at the N-terminus enhances autophagy while at the C-terminus inhibits. Akt is a major mediator of insulin signaling and has been reported to be involved in mediating obesity and type 2 diabetes-related inflammatory disease (116). Metformin inhibits Akt activating autophagy, which is consistent with its activation of autophagy by lowering G6P as a result of inhibition of glucose flux and glycolysis (117). Deletion of Akt promotes macrophage polarization towards to M1-MФs and increased NO synthesis from arginine (118, 119). These observations suggest that, besides its antidiabetic effect, metformin can significantly reduce the risk of TB in patients with diabetes mellitus (120). Contradictory findings on the relationship between glucose metabolism and autophagy have been also revealed. Collins and coworkers reported that loss of mTORC1 in macrophages enhanced pro-inflammatory functions which are normally related to M1-MФs with upregulated glycolysis and activation of mTORC1 (121). These results were evaluated using rapamycin to polarize mouse and human macrophage models (122). The discrepancy can be explained by the differential localization of mTOR in lysosomes under M1- and M2- conditions (119). Mechanistically, it is known that, under starvation of glucose, a p38 MAPK-dependent pathway can trigger autophagy independent of the AMPK-mTOR pathway (123). We illustrate a diagram of Mtb-killing or survival during autophagy or nitric oxide (NO) through metabolite-sensing kinases corresponding to glucose homeostasis (**Figure 3A**).

During MTB infection of macrophages, glucose metabolism plays a significant role centered around autophagy. Glucose is a major metabolic source producing ac-CoA through glycolysis and SAM through serine biosynthesis and one-carbon metabolism. Ac-CoA and SAM are the necessary cofactors of histone acetyltransferases and methyltransferases (including DNA methyltransferases); further, glycolysis consumes NAD^+^ that is an essential cofactor of histone deacetylases. It is evident that glucose metabolism controls the level of cofactors and thereby, the epigenetic regulation through histone acetylation and methylation (and DNA methylation) which is further reviewed below. IFN-γ which drives M1-MΦs promotes a metabolic switch from oxidative phosphorylation to glycolysis, a process similar to the Warburg effect of hypoxia in cancer cells. Increased glycolysis causes the production and enrichment of copious lactate. Interestingly, Zhang et al. identified that histones can be modified by lactylation, and increased lactate promoted histone lactylation and polarization towards M2-MΦs (124). These data suggest that M1-MΦs can self-differentiate into M2-MΦs after prolonged glycolysis culminating in excess lactate. Noe et al. also show that glucose is still required for M2-MΦ polarization; under glucose starvation, exogenously added lactate matching the measured concentration of lactate produced by IL-4 primed M2-MΦs rescued the loss of lactate endogenously produced from glucose metabolism. This process enriched citrate from pyruvate by the half-blocked TCA cycle, and subsequently increased ac-CoA after ACLY cleavage resulting in global histone acetylation and M2 gene promoter-specific acetylation (**Figure 3B**) (125). Together, these observations indicate that lactate is a driver of M2 polarization from either M0- or M1-MΦs. Interestingly, the lactate-treated M2-MΦs had increased capacity to kill MTB possibly through autophagy (94). However, it remains unclear how histone lactylation is regulated and whether it causes histone acetylation to promote autophagy during TB.

## Acetyl-CoA production from glycolysis is regulated by protein acetylation and sirtuins

Proteins acetylation dictates how cells choose glycolytic versus oxidative metabolism as a function of energy availability and then determine storage or utilization of carbon source (126, 127). Being a fundamental building block for fatty acid synthesis, ac-CoA is a necessary co-substrate of protein acetyltransferases to provide acetyl groups for acetylation of proteins, mostly on the ϵ-amino group of lysine, but also on the hydroxyl groups of serine, threonine, and tyrosine specifically among bacteria (128). Though it can be formed by fatty acid β-oxidation, amino acid catabolism, and break-up of citrate, ac-CoA is mainly produced by glycolysis (129, 130). Many enzymes in glycolysis, TCA cycle and proteins in mitochondria are the substrates of histone acetyltransferases whose acetylation sites have been identified by proteomics; nearly two-thirds of glycolic and TCA cycle enzymes show acetylation sites (14). Acetylation promotes or inhibits the activities of these enzymes, thereby increasing or decreasing the production of metabolites (129, 131). For instance, the enzymatic activity of phosphoglycerate mutase-1 (PGAM1), a protein critical for glycolysis, is regulated by glucose availability and SIRT1-dependent reversible deacetylation (15). When glucose is available, acetylation of PGAM1 stimulates catalysis. When glucose is restricted, SIRT1 levels increase, leading to deacetylation of PGAM1 and decrease in its enzymatic activity (15). Another positive correlation between acetylation and enzymatic activity is SIRT2 expression during iPSC reprogramming when OCT4 induces miR-200c-5p to suppress the expression of SIRT2 *via* microRNA binding sites in its coding sequence. As a result of downregulation of SIRT2, the activities of glycolytic enzymes (ALDOA, GAPDH, PGK1, ENO1 and PKM1/2) are increased due to elevated acetylation levels of these proteins (132). In contrast, acetylation of some glycolic enzymes can reduce their activity. It was reported that PKM2, a pyruvate kinase which is involved in the last step of glycolysis to produce pyruvate and ac-CoA, is acetylated at K305 by p300/(CREB binding protein) associated factor (PCAF) resulting in a decrease of its enzymatic activity (133). Moreover, acetylation of PKM2 enhanced its interaction with HSC70 and promoted its lysosome-dependent degradation *via* chaperone mediated autophagy under high glucose intake (133). Deacetylation at K305 by SIRT2 inhibits the pyruvate kinase of PKM2 by promoting its tetramerization (134), whereas deacetylation at K433 by SIRT6 inhibited the pyruvate kinase of PKM2 by suppressing its nuclear localization (135). The decrease of both enzymatic activity and protein level resulted in the accumulation of glycolytic metabolites upstream of PKM2, including FBP (fructose-1, 6-bisphophate) and G6P (glucose-6-phosphate). FBP was then found to couple with glycolytic flux to activate Ras and its downstream targets MEK and ERK driving autophagy (136); in contrast, G6P inhibited autophagy during glucose depletion (101, 102, 137). Interestingly, desuccinylation at K311 by SIRT5 counters acetylation at K355 and K433 to activate the pyruvate kinase of PKM2 by promoting its tetramer-to-dimer transition and nuclear localization, thereby blocking macrophage IL-1β production and preventing dextran sulfate sodium (DSS)-induced colitis in mice (138). These observations suggest that glucose metabolism and ac-CoA production are regulated by the acetylation states of glycolytic enzymes and sirtuin proteins play a major regulatory role.

## Histone acetylation is responsive to metabolite levels and regulates autophagy

Acetylation of histones is a critical epigenetic modification that changes chromatin architecture and regulates gene expression. Many studies show that metabolism regulates acetylation, and, the changes in glucose metabolism can regulate histone acetylation (12, 13, 139). Using multiplexed stable isotopic labeling by amino acids in cell culture (SILAC)-based proteomics, Locasale’s lab found that the acetylation levels of half of identified histone acetylation sites and lysine acylation modifications at these sites were modulated by the rate of glycolysis and that histone acetylation levels were strongly correlated with ac-CoA levels and inversely associated with the ratio of ac-CoA to free CoA (11). However, glycolysis-generated, ac-CoA-dependent histone acetylation was competitively regulated by citrate-generated ac-CoA by ATP-citrate lyase (ACLY) (140–142). Moreover, the production of ac-CoA seems to be counter-balanced by utilization of ac-CoA to form lactate from pyruvate *via* LDH, reaction with OAA to form citrate entering the TCA cycle, acetylation of amino acids, and synthesis of fatty acids and other molecules in various metabolic pathways. Therefore, histone acetylation regulates metabolism and macrophage activation, whereas acetylation is fine-tuned by metabolism in polarized macrophages (143, 144). LPS/IFN-γ promotes polarization towards M1-MΦs characterized by up-regulated glycolysis and production of pro-inflammatory cytokines, such as IL-1β whose expression is enhanced by histone acetylation (145). The acetylation was thought to be due to the increased production of ac-CoA from elevated glucose metabolism and upregulated ACLY that reciprocally up-regulates glycolytic gene expression (146, 147). Higher levels of histone acetyltransferase MOF expression and acetylation at histone H4K16 were detected in inflammatory macrophages at the wound sites of diet-induced-obese mice compared to the anti-inflammatory macrophages in the healing phase (148). In addition, ACLY-mediated citrate metabolism in the TCA cycle contributes to the production of ROS and RNS in inflammatory cells (149). In contrast, Noe and co-workers reported that ACLY activation also promoted naive M0 to M2 polarization through the lactate-citrate-ac-CoA route for histone acetylation in tumor microenvironments (TME) (125). It remains unclear whether data from animal studies can be translated to humans although, some studies do reveal a positive correlation. For example, Vlad et al. found that histone acetylation, the expression of histone acetyltransferases p300, and the expression of NADPH oxidase-5 (Nox5) were all elevated in human atherosclerotic specimens. They were co-localized in the area of CD45^+^/CD68^+^ immune cells and lipid-rich deposits within atherosclerotic plaques (150); in these microenvironments, increased glucose intake and enhanced glycolysis were proposed (151). Consistently, ACLY was activated in inflammatory macrophages and human atherosclerotic plaques (152). In contrast, inhibition or silencing of Slc25a1, a transporter of citrate, resulted in decreased production of NO, ROS, and PGE_2_ in U937 cells (153) and inhibition of ACLY had the same effects (154). However, the role of ACLY in macrophage polarization was challenged by Namgaladze et al. who found that silencing ACLY expression using CRISPR/Cas9 in human THP-1 cells did not attenuate IL-4 induced gene expression as ACLY inhibitors did and concluded that ACLY might not be the major regulator of nucleocytoplasmic ac-CoA contributing to IL-4-induced M2-MΦ polarization of human macrophages (155). Erika Palmier and coworkers performed ^13^C tracing experiments using [U-^13^C]-glucose and glutamine and found that NO inhibited mitochondrial aconitase (ACO2) resulting in blockade of TCA, and that inflammatory macrophages rerouted pyruvate away from pyruvate dehydrogenase (PDH) in an NO-dependent but hypoxia-inducible factor 1α (HIF1α)-independent manner. This process promoted glutamine-based anaplerosis which sustained the TCA cycle using the glutamine generated αKG and OAA from pyruvate carboxylation (80). This suggested that ac-CoA generated from glycolysis would be reduced resulting in decreased histone acetylation in M1-MΦs due to NO-mediated inhibition of PDH. This is an intriguing cross regulation by NO in M1-MΦs that needs additional investigation. Besides production of ac-CoA from metabolism, histone acetyltransferases themselves also determine the acetylation level of histones and expression of autophagy genes. Fullgrabe et al. demonstrated that induction of autophagy by starvation or rapamycin inhibition of mTOX was coupled to reduction of histone H4 lysine 16 acetylation (H4K16ac) through downregulation of the histone acetyltransferase hMOF/KAT8/MYST1 in both mouse embryonic fibroblasts (MEF) and human transfected cells (156). However, downregulation of histone acetylation and hMOF also led to a transcriptional repression of autophagy genes based on a feedback mechanism, preventing chronic autophagy that could lead to cell apoptosis (156).

## Sirtuins and NAD^+^ regulate protein/histone deacetylation and autophagy-mediated killing of bacteria

### Sirtuins and antimicrobial mechanisms

Sirtuins, the class III histone deacetylases (HDAC), are crucial regulators of inflammation and immune cell metabolism and function (157–159). Metabolism is controlled not only by histone acetylation but also deacetylation. Activities of sirtuins are dependent of NAD^+^, NADH, or their ratio as NAD^+^ is their essential co-substrate (160). There are seven currently known sirtuins (SIRT1-7). Each sirtuin isoform is located at a specific compartment of the cell and has its specific preferred substrate. SIRT1, SIRT6, and SIRT7 are predominantly located in the cell nucleus (161). SIRT1 also exists in cytosol and is a master metabolic regulator and the most studied sirtuin protein so far; it is downregulated in cells with high insulin resistance and its overexpression increases insulin sensitivity (162–164). High concentration of glucose significantly downregulates SIRT1 expression at both mRNA and protein levels, which is related to upregulation of pro-inflammatory cytokines, IL-1β and TNF-α in RAW264.7 macrophages (165). On the other hand, SIRT1 is up-regulated under calorie-restrict conditions known to extend life-span (166, 167). SIRT1 also stimulates autophagy by deacetylating autophagy-related proteins (*ATG*) including *ATG5, ATG7*, and *LC3* which are required for autophagy in cultured cells, embryonic and neonatal tissues (168, 169). SIRT1-dependent mechanism of autophagy induction is not clear; it may stabilize *ATG* proteins by forming a complex with them to prevent from degradation or prevent deacetylation at the promoters of *ATG5* and *ATG7* genes by other sirtuins due to its usage of NAD^+^ thereby activating expression of *ATG5* and *ATG7* (170). SIRT1 can also promote autophagy by activating AMPK to improve mitochondrial function (171), inhibiting the mTORC1 signaling pathway (172), and enhancing transcriptional activities of FOXO1 and FOXO3 through their deacetylation (169). Cheng and co-workers reported that MTB infection down-regulated SIRT1 in animal models and patients with active TB. Activation of SIRT1 by its activators, such as Resveratrol, not only induced autophagy but also dampened MTB-mediated chronic inflammation *via* deacetylation of RelA/p65 and impaired binding of RelA to the promoter of inflammatory genes (173). Similar results were obtained by others using mouse models (174). Another mechanism of the anti-TB property of SIRT1 was revealed by Yang, et al. who found that activation of SIRT1 prevented cell death in MTB-infected macrophages through BAX and GSK-3β (175, 176). In addition, SIRT1 activators also enhanced anti-TB drug efficacy (173). Interestingly, SIRT1 inhibition by sirtinol has also been reported to induce autophagy and autophagic cell death in MCF-7 cells (177). The mechanism is not known. Off target effects on NAD^+^ biosynthesis and/or salvage pathways is possible, since an enhanced activation of these pathways increases autophagy (178). SIRT6 is essentially a deacetylase of histones H3 and H4, which changes chromatin density and regulates gene expression and is required for normal base excision repair and double-strand break repair of DNA damage in mammalian cells (179). SIRT6, together with histone H3K9 methyltransferase G9a, participate in inflammatory response in macrophages, contribute to the IFN-sterol antiviral activity, and play an active role in inflammation-mediated glucose intolerance during obesity (180, 181). SIRT6 seems to facilitate MTB survival in macrophages by epigenetically modulating host cholesterol accumulation (182). SIRT7 was originally found to facilitate the transcription of DNA by DNA polymerase I, DNA polymerase II, and DNA polymerase III (183, 184). It has recently been found as a nutrient sensor similar to SIRT1 during glucose starvation or calorie-restricted diet and its depletion causes impaired activation of autophagy (185). The effects of SIRT7 on tuberculosis remain unclear.

SIRT2 is mainly cytoplasmic and also exists in nuclei where it can deacetylate histones. SIRT2 suppresses T cell metabolism by targeting key enzymes involved in glycolysis, TCA cycle, fatty acid oxidation, and glutaminolysis. SIRT2-deficient murine T cells and SIRT2 blockaded human tumor-infiltrating lymphocytes showed increased glycolysis and oxidative phosphorylation, enhanced proliferation and effector functions and thereby superior antitumor activity (186). SIRT2 dysregulated autophagy in high-fat-exposed mouse immune-tolerant and hypo-inflammatory macrophages (187). We found that the expression of SIRT2 was higher in MTB-infected human peripheral blood derived M2-MΦs which had lower autophagy activity than M1-MΦs infected with MTB (6). Pharmaceutical inhibition of SIRT2 increased autophagy and killing of MTB by M2-MΦs; morover, SIRT2 blockade combined with anti-TB drug dramatically increased MTB clearance in macrophages (6) (our unpublished data). Although Cardoso, et al. claimed that SIRT2 blockade only had a transient effect on MTB infection of mice (188), it is likely that human and mouse macrophages differ in sirtuin dependent regulation.

SIRT3, SIRT4, and SIRT5 are all found in the mitochondrial compartment and therefore implicated in regulating metabolic processes by deacetylating mitochondrial proteins. SIRT3 showed anti-inflammation property and mitigated endotoxin-induced acute lung injury (189). In MTB-infected macrophages, SIRT3 is down-regulated resulting in reduced expression of SIRT3-target genes including IDH2 and ETC complex I subunits and consequent accumulation of isocitrate, reduction of ETC complex I and II activity, lower GSH/GSSG ratio, and increase mtROS, promoting cell death (190). Paradoxically, activation of SIRT3 is necessary for autophagy and can provide protection for mitochondria in MTB-infected macrophages (191). However, anti-TB activity of SIRT3 is dependent on its genetic variants; for example, the minor allele genotype (A carriers) of rs3782118 shows a decreased risk of TB susceptibility, whereas the haptotype AGAAG (containing the major allete G of rs3782118) is associated with an increased risk of TB (192). SIRT4 is a mitochondrial ADP-ribosyltransferase that inhibits mitochondrial glutamate dehydrogenase 1 (GLUD1) activity, thereby downregulating insulin secretion in response to amino acids (193). SIRT4 shows opposite activity of SIRT1 and SIRT3 (194) and it counters SIRT1 and SIRT3 activity by suppressing their expression by rebalancing glycolysis and glucose oxidation during recovery of acute inflammatory response in monocytes (195).

SIRT5 exhibits multiple enzymatic activities, as it is a deacetylase, desuccinylase, and demalonylase, and capable of removing acetyl, succinyl, and malonyl groups from the lysine residues of proteins (196, 197). SIRT5 has dual functions of increasing ammonia production *via* promoting glutaminolysis and removing it by activating urea cycle. SIRT5 deacetylates and regulates carbamoyl phosphate synthetase (CPS1), the rate-limiting and initiating step of the urea cycle in liver mitochondria and therefore plays a critical role in ammonia detoxification (14, 197). On the other hand, SIRT5 stabilizes glutaminase (GLS) by desuccinylation, the enzyme transforming glutamine into glutamate generating ammonia (198). As ammonia is a diffusible regulator of autophagy (199), the regulation of autophagy by SIRT5 may be dependent on net ammonia concentration produced and consumed from glutaminolysis and urea cycle. Indeed, Polletta et al. demonstrated that in human breast cancer MDA-MB-231 and mouse myoblast C2C12 cell lines, ammonia production was increased when SIRT5 was silenced and decreased in SIRT5-overexpression cells (200). Morover, when GLS was activated by SIRT5, production of ammonia was increased and consequently autophagy activity was increased, whereas inhibition of SIRT5 decreased both ammonia production and autophagy (200). SIRT5 is therefore appears to be a potential regulator of autophagy and has additional, tangential effects like desuccinylation of mitochondrial proteins (201). Desuccinylation of ETC complex I and II occurs upon the binding of SIRT5 to the mitochondria-exclusive phospholipid-cardiolipin, which maintains the integrity of ETC residing on the inner mitochondrial membrane hence promoting the oxidation of NADH into NAD^+^ and the production of ROS and ATP (202, 203). Further, SIRT5 can desuccinylate glycolytic enzyme PKM2 causing its deactivation; in LPS activated but SIRT5 knock-out macrophages, IL-1β production was boosted due to an increase in succinylation of PKM2, demonstrating that SIRT5 is related to anti-inflammation (138). In our studies, we found that SIRT5 was up-regulated in MTB-infected and -uninfected human M1-MΦs in contrast to SIRT2 which was up-regulated in M2-MΦs (6). We found that both inflammatory IL-1β production and autophagy were up-regulated in MTB infected M1-MΦs unlike mouse macrophages (6, 96), and in contrast with Wang, et al. (138), we found that SIRT5 was related to a pro-inflammatory response. These issues underscore sirtuin- dependent differences between human and mouse macrophages. In cancer studies, SIRT5 was found to be downregulated in gastric cancer tissues and it enhanced autophagy *via* the AMP-activated protein kinase-mTOR signaling pathway (204). From these observations, we propose a tentative conclusion, though debatable, that of the seven sirtuin proteins, SIRT1, 3, 5, and 7 perform a protective function against infections with MTB whereas, SIRT2, SIRT4 and SIRT6 interfere with macrophage pathways facilitating pathogen survival. However, it is also likely that sirtuins are interdependent and compete with the shared resource of NAD^+^; for example, activity of one sirtuin protein may be enhanced by inhibition of another one. An example is that SIRT5 counters the inhibitory effects of SIRT2 and enhances the innate immune responses in macrophages by blocking SIRT2-dependent deacetylation of RelA/p65 activating NF-γB and increased production of downstream cytokines (205).

### Sirtuins and arginine metabolism

**Figure 4:**
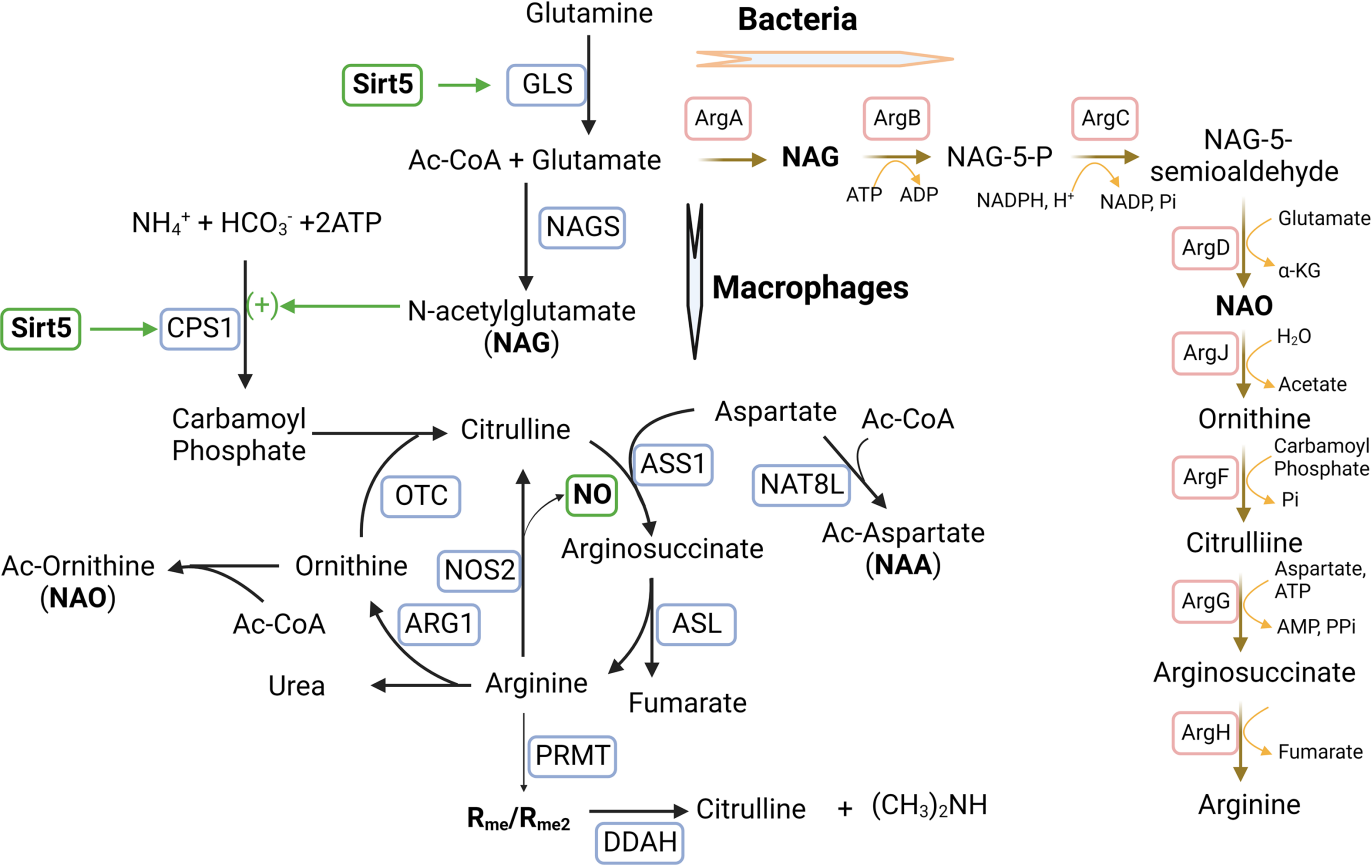
Sirtuin5 plays a vital role in arginine metabolism during macrophage activation and polarization. Arginine is converted into citrulline to release NO in M1-MΦs where iNOS/NOS2 is up-regulated, whereas arginine is converted into ornithine in M2-MΦs where ARG1 is up-regulated. In addition, arginine metabolism is regulated by glutamine metabolism which is involved in the urea cycle by N-acetylglutamate (NAG). NAG which is an allosteric activator and is required for the initial and rate-limiting enzyme of the urea cycle, carbamoyl phosphate synthetase 1 (CPS1). The formation of this unique co-substrate from glutamate and acetyl Coenzyme-A is catalyzed by NAG synthase (NAGS). Sirtuin-5 (SIRT5) desuccinates and activates CPS1 to promote the formation of carbamoyl phosphate from ammonia. Carbamoyl phosphate can modify ornithine to form citrulline through the enzyme ornithine transcarbomoylase (OTC). Citrulline can react with aspartate facilitated by the catalytic enzyme arginosuccinate synthetase (ASS1) to form arginosuccinate, which can return to arginine and fumarate through argininosuccinate lyase (ASL). Both aspartate and ornithine can be acetylated to form acetylated aspartate (NAA) and acetylated ornithine (NAO). Asymmetric di-methylated arginine (ADMA/R_me2_) can be hydrolyzed by enzyme dimethylarginine dimethylaminohydrolase (DDAH) into citrulline and dimethylamine. In bacteria, arginine biosynthesis can start with glutamate acetylation and a set of bacterium-specific catalytic enzymes (ArgA-H) are involved. *Additional Symbols*: NAT8L, N-acetyltransferase 8 like; PRMT, Protein arginine methyltransferase.

An intriguing effect of SIRT5 is its ability to regulate arginine metabolism and NO production. As discussed above, SIRT5 deacetylates, desuccinates, and deglutarylates CPS1 to promote the formation of carbamoyl phosphate from ammonia in the urea cycle (196, 197). This process potentially increases the synthesis of citrulline because of interaction between carbmoyl phosphate and ornithine (**Figure 4**). Interestingly, acetylated glutamate (NAG) additively activates CPS1 (206). With the aid of catalytic enzyme arginosuccinate synthetase (ASS1), citrulline reacts with aspartate to form arginosuccinate which is then converted into arginine and fumarate by argininosuccinate lyase (ASL). Both ornithine and aspartate can be acetylated in macrophages. Therefore, it appears that acetylation of amino acids (glutamate, aspartate, and ornithine) and SIRT5 are involved in the conjugated urea cycle and arginine metabolism cycle. Nitric oxide, the RNS (reactive-nitrogen-species) precursor, is produced by arginine oxidation with the help of iNOS/NOS2. Increased citrulline can replenish arginine consumption for oxidation (207). We propose that an identification of the targets and functions of SIRT5 using mouse liver and human kidney cells can shed a light on the role of SIRT5 during macrophage activation and polarization.

In this direction, we measured mRNA expression of SIRT5 which was significantly higher in MTB-infected and uninfected M1-MФs than in M2-MФs cultured under identical conditions (6). Because we had detected that a majority of the proteins in the ETC complex I in M1-MФs was up-regulated (86), we suspected that not only desuccinylation by SIRT5 but also protein expression of ETC complex I promote NADH oxidation into NAD^+^ and ROS in M1-MФs. We also found an inverse relationship between acetylated amino acids and acetylated histones (86). Therefore, we speculated that acetylation of amino acids and acetylation of histones might compete for ac-CoA to fulfill acetylation; in M1-MФs, glycolysis generated acetyl-CoA cannot enter the partially blocked TCA cycle but is consumed by acetylation of amino acids as a consequence of which, the supply of ac-CoA for acetylation of histones is diminished. Another possibility is that histone acetylation was reduced by deacetylation with increased production of NAD^+^ by ETC complex I (86). Interestingly, acetylated aspartate (NAA), glutamate (NAG) and ornithine (NAO) were not only enriched in M1-MФs but were also connected with arginine metabolism (**Figure 4**). Both NAO and methylated arginine inhibit iNOS/NOS2 required for the production of NO (208–211). In addition, arginine is metabolized into citrulline releasing NO to form RNS that is upregulated in M1-MФs. These intriguing data led us to the tantalizing questions: how does SIRT5 regulate acetylation of amino acids and histones to leverage arginine metabolism and further, how is RNS production regulated by SIRT5 *via* arginine metabolism?

We note here that, bacteria including Mtb can synthesize arginine from glutamate by acetylation. NAG which is synthesized from glutamate by ArgA and NAO which is synthesized from NAG-5-semialdehyde by ArgD, are the important intermediates. Because mutation dependent loss of function for ArgA or ArgD led to antibiotic resistance in bacteria (212), it appears important to determine, how amino acid acetylation in macrophages is regulated by SIRT5 to replenish NAG and NAO during urea and arginine cycles in relation to drug resistance. Additional studies are warranted in this area.

### Sirtuins and tryptophan metabolism

**Figure 5:**
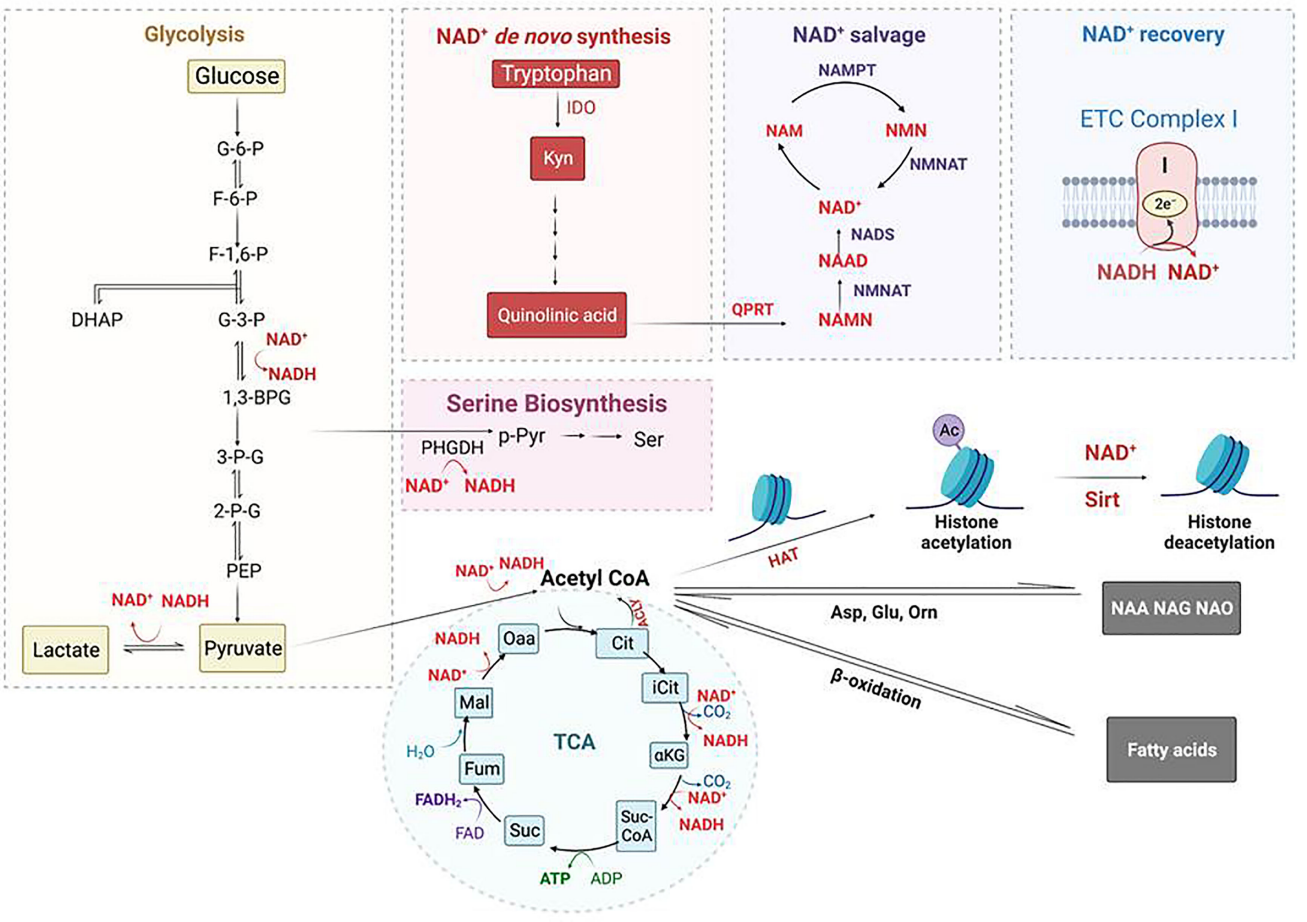
Regulation of Histone acetylation and deacetylation by metabolism-generated acetyl-CoA and NAD^+^. Acetyl-CoA is an essential co-substrate of histone acetyltransferase (HAT) that is mainly generated from glycolysis and fatty-aid β-oxidation; it is required for histone acetylation, amino acid acetylation including forming n-acetyl-aspartate (NAA), n-acetyl-glutamate (NAG), and n-acetyl-ornithine (NAO), and fatty-acid synthesis. NAD^+^ is an essential co-substrate of NAD^+^-dependent histone deacetylases that includes Sirtuin proteins. NAD^+^ is consumed by glycolysis (2 molecules) and TCA cycle (3 molecules), whereas it is regenerated from NADH oxidation *via* conversion of pyruvate into lactate and through ETC complex I. NAD^+^ is biosynthesized from quinolinic acid, the end product of tryptophan metabolism, catalyzed by the rate-limiting enzyme quinolinate phosphoribosyl transferase (QPRT). The NAD^+^ *de novo* biosynthesis pathway is coupled with and regulated by the NAD^+^ salvage pathway. Regulation of NAD^+^ usage and production in MΦs controls Sirt deacetylase activity, and hence, histone acetylation level. *Additional Symbols*: ACLY, ATP-citrate lyase; NAM, niacinamide; NAMPT, nicotinamide phosphoribosylransferase; NMN, nicotinamide mononucleotide; NMNAT, nicotinamide nucleotide adenylyltransferase; Orn, ornithine; PHGDH, phosphoglycerate dehydrogenase; p-Pyr, phosphopyruvate.

Deacetylation activities of sirtuins are regulated by the availability of NAD^+^. Two and three molecules of NAD^+^ are respectively consumed in glycolysis and TCA cycle. NAD^+^ can be recovered from NADH oxidation, pyruvate reduction to lactate, and the redox reaction in ETC complex I. NAD^+^ can also be *de novo* synthesized from tryptophan metabolism and synthesized *via* the nicotinamide salvage pathway (**Figure 5**). Thus, the overall level of NAD^+^ is well regulated under physiologic conditions to maintain optimal metabolism, appropriate energy production, and proliferation. Isotope tracing studies performed by Minhas et al. revealed that macrophage NAD^+^ was derived substantially from kynurenine pathway of tryptophan metabolism to maintain normal innate immune functions, whereas breakdown of this *de novo* NAD^+^ synthesis pathway could occur after LPS stimulation. They also demonstrated that inhibiting the expression of quinolinate phosphoribosytransferase (QPRT) decreased NAD^+^ level and caused innate immune dysfunction during aging and age-related diseases (213). Another study by Cameron et al. indicated that synthesis of NAD^+^ by the salvage pathway drove an immediate macrophage inflammatory response to LPS (214). The mechanistic insight was that LPS-induced ROS caused DNA damage through heightened expression of CD38 and increased PARP activity, a process which consumes NAD^+^ to trigger the salvage pathway for repletion of NAD^+^ (214, 215).

Using triomics to analyze IFN-γ activated but rested and uninfected human donor derived M1-MΦs, we found significantly increased expression of QRPT and the production of Niacin (aka, nicotinic acid or vitamin B_3_) which is the precursor of NAD^+^; this indicated up-regulated *de novo* NAD^+^ synthesis through tryptophan metabolism. We proposed that elevated NAD^+^ level would result in an increased deacetylation by sirtuins and thereby decreased histone acetylation. Indeed, we found decreased histone acetylation in uninfected M1-MΦs using mass spectrometric measurements (86). However, during MTB infection, we propose that NAD^+^ level could be depleted by glycolysis or the inhibition of NAD^+^ salvage pathway by tuberculosis necrotizing toxin (TNT) resulting in the death of macrophages (216, 217). Further, NAD^+^ replenishment alone or its combination with resveratrol (RSV) or cyclosporin A (CsA) can counter the toxicity of TNT and protect macrophages from MTB-induced cell death (173, 216, 218). Others found that NAD^+^ levels can also be raised by treatment with fatty acid oxidation inhibitors such as Trimetazidine (TMZ) which induced NADPH oxisase and autophagy mediated control of tuberculosis (219). Together, these data suggest that cellular NAD^+^ concentration controls both sirtuin deaceylase activity and antimycobacterial function of macrophages.

### Pharmacological modulation of sirtuins to increase antimicrobial mechanisms

Sirtuins have been found as potential immunotherapeutic targets against tuberculosis because of their regulation of central energy metabolism *via* NAD^+^-dependent deacetylation. It has been reported that MTB infection depleted NAD^+^ level and perturbed sirtuin activity in MΦs (173, 190, 191, 217). Others reported that inhibition of SIRT2 with AGK2 restricted the growth of both dug-sensitive and -resistant strains of MTB and enhanced the efficacy of anti-TB drug Isoniazid in the mouse model of infection (220). In contrast, SIRT1 activators, such as resveratrol (RES), achieved a similar outcome by reducing lung pathology, chronic inflammation, and enhanced the efficacy of anti-TB drugs (173). As previously noted, hMOF is a specific histone H4K16 acetyltransferase; low activity of hMOF and low H4K16 acetylation is related to starvation-induced autophagy, which causes chronic repression of autophagic genes (156). SIRT1 is a H4K16 specific deacetylase. Mechanistically, activation of SIRT1 may keep the global H4K16 acetylation at low levels but on the other hand, it may deacetylate and activate ac-coA synthetase 1 (AceCS1) accumulating ac-CoA from acetate (221). Moreover, SIRT1 can also deacetylate hMOF to facilitate its binding to the chromatin at the promoters of autophagic genes promoting H4K16 acetylation due to increase in AceCS1 derived ac-Co-A (222). In murine J2-macrophages, the mRNA expression levels of SIRT1, SIRT3, SIRT5, and SIRT7 were all decreased at 24 hr post-infection of TB, which was also validated using mouse bone marrow derived macrophages (BMDM) (190). A detailed study of SIRT3 demonstrated that, over-expression of SIRT3 or treatment with SIRT3 activator Honokiol prevented MTB from inducing mitochondrial ROS accumulation in murine BMDM and cell death, whereas reduced expression of SIRT3 in Sirt3^-/-^ mice increased bacterial burden (190). A similar report revealed that SIRT3 enhanced anti-TB defense through coordinated mitochondrial and autophagic functions (191). SIRT7 has protective effects against TB-infection through regulation of NO production and apoptosis demonstrated using an *in-vitro* model (223). Prakhar et al. observed restricted growth of TB and development of granulomatous lesion in the lungs and spleen of SIRT6 heterozygous mice infected with TB (182). Together these data suggest that the activators of SIRT3, SIRT5 and SIRT7 are potential anti-TB drugs in addition to the SIRT1 activator-Resveratrol. In contrast, we found that SIRT2 blockade increases autophagy-mediated killing of MTB. Of note, there are no data on whether SIRT4 contributes to anti-tuberculosis immunity.

### Prospects for sirtuin modulators as drugs against tuberculosis

Despite reports that sirtuin inhibitors or activators in combination with the FDA-approved frontline anti-TB drugs enhance killing of drug resistant and dormant TB (173, 220, 224), none has been approved by FDA. Metformin is a direct SIRT1 activator based on computational modeling and experimental validation (225). Although it is not a TB-specific drug, it shows therapeutic efficacy for patients who have comorbidity of TB and diabetes and can be used as a pure adjunctive therapy for TB (226). Because, small chemical compounds that modulate sirtuin function have been pursued as anticancer agents (227), we propose that efforts should be made to use a combination of sirtuin activators and inhibitors to treat tuberculosis in combination with existing therapies.

Beside sirtuin proteins, the NAD^+^ biosynthesis pathway may also be a promising target for tuberculosis therapy. Recent elucidation of the mechanism of isoniazid (INH), a frontline anti-TB drug, indicated that INH couples with NADH catalyzed by KatG to form the active INH-NAD adduct, which in turn, binds tightly to the enoyl-acyl carrier protein reductase InhA so that the synthesis of mycolic acid for mycobacterial cell wall formation is inhibited (228). As MTB depends solely on its own *de novo* pathway to meet its NAD^+^ demand (229), MTB-QPRT provides an attractive target for designing novel anti-TB drugs (230). Coincidentally, NAD^+^ in the host M1-MΦ is significantly higher than M2-MΦs to maintain autophagy and bactericidal activity. Because of QPRT occurs in both host macrophages and MTB, its non-specific inhibition would decrease autophagy mediated killing capacity of macrophages. As crystal structures of both human and MTB derived QPRT have been elucidated (229, 231), to avoid toxicity, a drug to selectively target MTB-QPRT but not human-QPRT based on their structural difference at the substrate binding sites would be crucial. Quinolinic acid (QA) is the first intermediate in the *de novo* pathway of NAD^+^ biosynthesis that is common to all organisms and is mainly produced by the degradation of tryptophan in most eukaryotes. In contrast, in prokaryotes, including MTB, it is mainly produced from l-aspartate and dihydroxyacetone phosphate by the enzymes encoded by *nadA* (quinolinic acid synthetase) and *nadB* (l-aspartate oxidase) (232). Therefore, we propose that a drug to target nadA/B may be an alternative to QPRT inhibitors to control tuberculosis (233).

## Sirtuins intersect the serine biosynthesis, one-carbon metabolism, and methylation of DNA and histones

**Figure 6:**
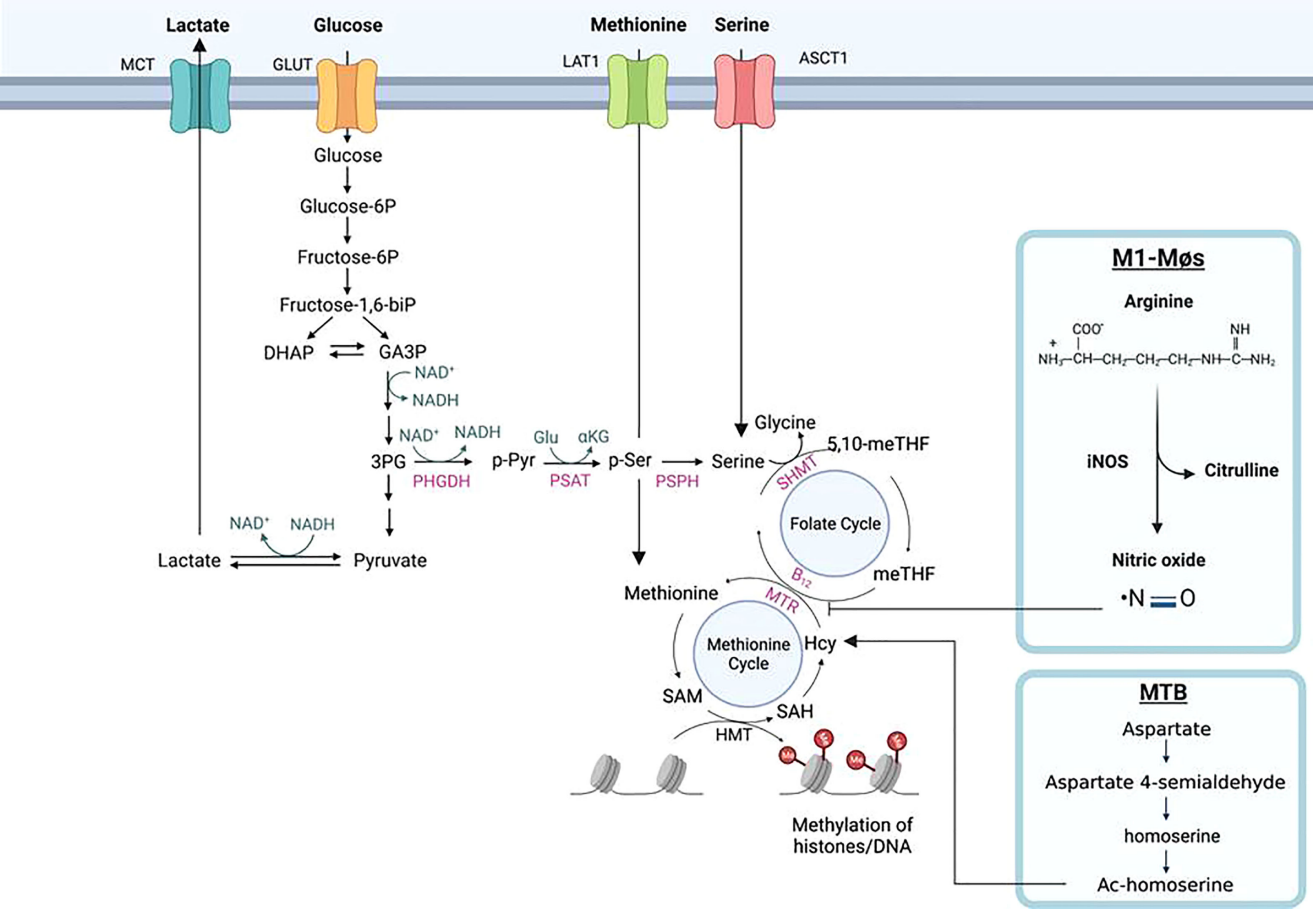
Histone methylation through one-carbon metabolism and serine biosynthesis in macrophages. Serine is biosynthesized from 3-phophoglycerol (3PG), an intermediate of glycolysis, by phosphoglycerol dehydrogenase (PHGDH) to form phosphopyruvate (p-Pyr) and catalyzed by phosphoserine aminotransferase (PSAT) to form phosphoserine (p-Ser) and then phosphoserine phosphate (PSPH) to form serine. With the aid of catalytic enzyme serine hydroxymethyltransferase (SHMT), serine is further converted into glycine donating one-carbon (a methyl group) to the tetrahydrofolate (THF) in the folate cycle to form sequentially 5, 10-methylenetetrahydrofolate (5,10-meTHF) and 5-methyl-tetrahydrofolate (meTHF); the methyl group of the latter is transferred to homocysteine (Hcy) to form methionine (Met) and S-adenosyl-methionine (SAM). SAM is the co-substrate of methyltransferases for DNA and histone methylation. Methionine and serine can also be respectively delivered from extracellular environment to the cells by their transporters, L-type amino acid transporter/solute carrier family member 5 (LAT1/SLC7A5) and alanine/serine/cysteine/threonine transporter 1 (ASCT1). The methyl transfer from meTHF to Hcy needs methionine synthesis (MS/MTR) and its co-substrate vitamin B_12_. In M1-MΦs, elevated nitric oxide (NO) poisons vitamin B_12_ causing deactivation of MTR and the disruption of one-carbon metabolism, resulting in reduced Met and SAM for histones/DNA methylation. In *Mycobacterium tuberculosis* MTB) infected MΦs, independent of vitamin B_12_, the pathogen can bypass the one-carbon metabolic pathway to synthesize methionine and SAM through homoserine, a product of aspartate metabolic pathway.

The biosynthesis of serine starts with the oxidation of 3-phosphoglycerate (an intermediate from glycolysis) by NAD^+^ to 3-phosphohydroxypyruvate and NADH catalyzed by phosphoglycerate dehydrogenase (PHGDH), which is a rate-limiting enzyme (**Figures 5**, **6**); the other two are Phosphoserine aminotransferase (PSAT) and Phosphoserine Phosphatase (PSPH). Since NAD^+^ is required for facilitating the functions of both PHGDH in serine biosynthesis and GAPDH in glycolysis, serine biosynthesis competes with the glycolysis pathway. Supporting this concept, serine deprivation in LPS-Simulated macrophages caused a reduction of pyruvate, decreased NAD^+^/NADH ratio, and decreased ROS level, partially resembling M2-MΦ phenotype but still maintaining a pro-inflammatory cytokine profile of M1-MΦs (234). However, Rodrigues et al. reported that serine is required for LPS induction of IL-1β mRNA expression but not inflammasome activation, because serine is used for conversion to glycine that is needed for macrophage GSH synthesis to support IL-1β production (235). Serine is required for the growth of MTB (236). Serine is converted to glycine by SHMT1 in the cytosol and SHMT2 in the mitochondria, which then donates one carbon to the folate cycle adjacent to the methionine cycle through methionine synthase (MTR) that in turn, requires vitamin B_12_ as a co-substrate. In the methionine cycle, SAM is synthesized from S-Adenosyl Homocysteine (SAH) with the donation of a methyl group from methionine. SAM is an essential co-substrate of methyltransferases, and provides the methyl group for methylation of histone, DNA and other biological compounds in the cells. In M1-MΦs, up-regulated glycolysis would increase the supply of 3-phosphohydroxypyruvate for serine biosynthesis. Because nitric oxide in M1-MΦs is toxic to vitamin B_12_, the transportation of B_12_ crossing the cell membrane is inhibited by hypoxia, and the mitochondrial citramalyl-CoA lyase (CLYBL) appears to be indirectly involved in the inhibition of vitamin B_12_ metabolism, depletion of B_12_ and as expected, subsequent inactivation of methionine synthase (MTR). As a result, one-carbon metabolism is hindered resulting in reduced formation of SAM and consequently, decreased methylation of histones or DNA (**Figure 6**). However, increased extracellular methionine uptake can still be triggered *via* the feedback mechanism to restore the loss. Excess methionine increases the production of SAM and DNA methylation attenuating LPS-induced inflammation (237). Because hypermethylation in macrophages reduces pro-inflammatory responses, we propose that a similar mechanism may favor the survival of MTB (238). Notably, MTB synthesizes its own methionine and SAM from homoserine which is produced through aspartate pathway (239, 240). Dinardo et al. performed methylation-sensitive enzyme-quantitative PCR (MSRE-PCR) and observed that in the PBMCs of TB-infected patients, pro-inflammatory genes including IL-1β and IFN-γ were DNA-hypermethylated resulting in dampened host immune responsiveness (4). MTB mediated hypermethylation of inflammatory genes is therefore a pathogen evasion strategy.

Proteomics has identified that the one-carbon enzyme, MTHFD1L (methylenetetrahydrofolate dehydrogenase [NADP^+^ dependent 1-like]) in the folate cycle, is a substrate for SIRT5 mediated desuccinylation/malonylation and SIRT5 also interacts with SHMT2 (241–244). In SIRT5 knock-down (KD) melanoma cells, reduced H3K4me3 and H3K9me3 were observed, indicating that reduced SAM production from impaired one-carbon metabolism of H3K4me3 and H3K9me3 sense the SAM levels in the cells (243, 245). SIRT5 also desuccinylates and activates SHMT2 to promote one-carbon metabolism and potential histone methylation in cancer cells (244). If this is true in immune cells, one-carbon metabolism and histone methylation would be enhanced in M1-MΦs as SIRT5 is up-regulated based on our RNA-seq data (6), which is opposite to what we proposed: that one-carbon metabolism would be down-regulated due to B_12_ depletion/MTR inactivation discussed above. In contrast, in breast cancer, SIRT2 regulates the reversible acetylation of PHGDH through TIP60 and promotes the binding of PHGDH and RNF5 to induce PHGDH degradation and reducing serine and glycine derived from glucose metabolism *via* the serine biosynthesis pathway in (246). If this information is also true in immune cells, in M2-MΦs upregulated SIRT2 would reduce serine synthesis from glucose metabolism potentially resulting in histone hypomethylation. Therefore, we propose that the methylation state in M1-MΦs versus M2-MΦ depends on which metabolic pathway is dominant- glycolysis and glucose intake, serine biosynthesis and intake, one-carbon metabolism and methionine intake, depending upon specific tissue microenvironments.

In order to understand how MTB regulates lysine and arginine methylation or other free amino acids and histones differently in M1- versus M2-MΦs, we will need to use isotope tracers and mass spectrometry. This will allow us to monitor how methyl migration to lysine and arginine residues from methionine/SAM produced by glucose derived serine occurs, and to determine whether serine is synthesized from intracellular source or directly taken up from extracellular nutrients in naïve versus polarized macrophages. Additionally, using isotope-labeled aspartate, we may be able to trace the methyl group migrating through the aspartate-homoserine-homocysteine route to lysine and arginine in MTB infected macrophages (**Figure 6**). We will then have a clear picture of methylation and epigenetic profiles differentially affected by metabolism in naïve or polarized macrophages infected with MTB.

## Conclusion and perspectives

Glycolysis not only generates energy (ATP) to meet the demand of cells for their surviving but also controls the homeostasis of NAD^+^ which prevents cells from death and is an essential co-substrate of sirtuin proteins, the type-III histone deacetylases. Importantly, glycolysis is also a source of directly or indirectly producing ac-CoA and SAM, the co-substrates of histone acetyltransferases and methyltransferases respectively. Upregulated glycolysis in M1-MΦs generates increased ac-CoA from pyruvate and thereby increased histones acetylation which is counter-regulated by NAD^+^. NAD^+^ is consumed in glycolysis and TCA cycle and other redox processes. It is also reproduced by oxidation in metabolic pathways such as lactate synthesis from pyruvate and ETC. Increased NAD^+^ from *de novo* synthesis and the NAD^+^ salvage pathway would tip the balance towards hypoacetylation. Glycolysis also links to serine biosynthesis, a fuel for one-carbon metabolism, and the synthesis of SAM for histone/DNA methylation. Metabolic switch between M1- and M2-MΦs therefore causes an imbalance of co-substrates (ac-CoA and SAM) of histone acetyltransferases and methyltransferases thereby changing the landscapes of acetylation and methylation of histones and proteins in the metabolic pathways. Consequently, metabolism controls macrophage gene expression, the production of anti-mycobacterial oxidants, and autophagy during pathogen infection. Since the co-substrates produced by metabolites from glucose are regulated by other metabolic pathways, future work needs to be focused on the dynamic correlation between metabolism and histone modifications through measurement of the levels of co-substrates produced in polarized macrophages and the states of histone modifications on a time scale. For example, we can use stable isotope labeled glucose as the major probe during early and late phase of infection. It is also important to seek an insight into the impact of glucose metabolism on the expression of cytokines and autophagy genes regulated by co-substrates. Moreover, we can use stable-isotope labeled glutamine and arginine, to probe the mechanism of how sirtuin proteins control glutaminolysis and NO production through conjunction of the urea cycle and arginine metabolism cycle. Sirtuin proteins and their substrates are therefore promising targets for treatment of tuberculosis and likely other intracellular infections.

## Author contributions

KZ wrote the manuscript. MS, EC, VS, BR, AK: contributed to supporting data and made graphics. CJ: Proposed the contents and edited the manuscript. All authors contributed to the article and approved the submitted version.

## References

[1] Rahlwes KC Dias BRS Campos PC Alvarez-Arguedas S Shiloh MU. Pathogenicity and virulence of mycobacterium tuberculosis. Virulence (2023) 14:2150449. doi: 10.1080/21505594.2022.2150449 36419223 PMC9817126

[2] Pal R Bisht MK Mukhopadhyay S. Secretory proteins of mycobacterium tuberculosis and their roles in modulation of host immune responses: Focus on therapeutic targets. FEBS J (2022) 289:4146–71. doi: 10.1111/febs.16369 35073464

[3] Rahman A Srivastava SS Sneh A Ahmed N Krishnasastry MV. Molecular characterization of tlyA gene product, Rv1694 of mycobacterium tuberculosis: A non-conventional hemolysin and a ribosomal RNA methyl transferase. BMC Biochem (2010) 11:35. doi: 10.1186/1471-2091-11-35 20854656 PMC2954847

[4] DiNardo AR Rajapakshe K Nishiguchi T Grimm SL Mtetwa G Dlamini Q. DNA Hypermethylation during tuberculosis dampens host immune responsiveness. J Clin Invest (2020) 130:3113–23. doi: 10.1172/JCI134622 PMC7260034 32125282

[5] Duan L Yi M Chen J Li S Chen W. Mycobacterium tuberculosis EIS gene inhibits macrophage autophagy through up-regulation of IL-10 by increasing the acetylation of histone H3. Biochem Biophys Res Commun (2016) 473:1229–34. doi: 10.1016/j.bbrc.2016.04.045 27079235

[6] Khan A Zhang K Singh VK Mishra A Kachroo P Bing T. Human M1 macrophages express unique innate immune response genes after mycobacterial infection to defend against tuberculosis. Commun Biol (2022) 5:480. doi: 10.1038/s42003-022-03387-9 35590096 PMC9119986

[7] Katada S Imhof A Sassone-Corsi P. Connecting threads: Epigenetics and metabolism. Cell (2012) 148:24–8. doi: 10.1016/j.cell.2012.01.001 22265398

[8] Freemerman AJ Johnson AR Sacks GN Milner JJ Kirk EL Troester MA. Metabolic reprogramming of macrophages: Glucose transporter 1 (GLUT1)-mediated glucose metabolism drives a proinflammatory phenotype. J Biol Chem (2014) 289:7884–96. doi: 10.1074/jbc.M113.522037 PMC3953299 24492615

[9] Viola A Munari F Sanchez-Rodriguez R Scolaro T Castegna A. The metabolic signature of macrophage responses. Front Immunol (2019) 10:1462. doi: 10.3389/fimmu.2019.01462 31333642 PMC6618143

[10] Soto-Heredero G Gomez de Las Heras MM Gabande-Rodriguez E Oller J Mittelbrunn M. Glycolysis - a key player in the inflammatory response. FEBS J (2020) 287:3350–69. doi: 10.1111/febs.15327 PMC7496292 32255251

[11] Cluntun AA Huang H Dai L Liu X Zhao Y Locasale JW. The rate of glycolysis quantitatively mediates specific histone acetylation sites. Cancer Metab (2015) 3:10. doi: 10.1186/s40170-015-0135-3 26401273 PMC4579576

[12] Friis RM Wu BP Reinke SN Hockman DJ Sykes BD Schultz MC. A glycolytic burst drives glucose induction of global histone acetylation by picNuA4 and SAGA. Nucleic Acids Res (2009) 37:3969–80. doi: 10.1093/nar/gkp270 PMC2709565 19406923

[13] Moussaieff A Rouleau M Kitsberg D Cohen M Levy G Barasch D. Glycolysis-mediated changes in acetyl-CoA and histone acetylation control the early differentiation of embryonic stem cells. Cell Metab (2015) 21:392–402. doi: 10.1016/j.cmet.2015.02.002 25738455

[14] Nakayasu ES Burnet MC Walukiewicz HE Wilkins CS Shukla AK Brooks S. Ancient regulatory role of lysine acetylation in central metabolism. mBio (2017) 8. doi: 10.1128/mBio.01894-17 PMC5705920 29184018

[15] Hallows WC Yu W Denu JM. Regulation of glycolytic enzyme phosphoglycerate mutase-1 by Sirt1 protein-mediated deacetylation. J Biol Chem (2012) 287:3850–8. doi: 10.1074/jbc.M111.317404 PMC3281715 22157007

[16] Gaal Z Csernoch L. Impact of sirtuin enzymes on the altered metabolic phenotype of malignantly transformed cells. Front Oncol (2020) 10:45. doi: 10.3389/fonc.2020.00045 32117717 PMC7033489

[17] Barron JT Gu L Parrillo JE. NADH/NAD redox state of cytoplasmic glycolytic compartments in vascular smooth muscle. Am J Physiol Heart Circ Physiol (2000) 279:H2872–2878. doi: 10.1152/ajpheart.2000.279.6.H2872 11087243

[18] Luengo A Li Z Gui DY Sullivan LB Zagorulya M Do BT. Increased demand for NAD(+) relative to ATP drives aerobic glycolysis. Mol Cell (2021) 81:691–707 e696. doi: 10.1016/j.molcel.2020.12.012 33382985 PMC8315838

[19] Locasale JW. Serine, glycine and one-carbon units: Cancer metabolism in full circle. Nat Rev Cancer (2013) 13:572–83. doi: 10.1038/nrc3557 PMC3806315 23822983

[20] Maddocks OD Labuschagne CF Adams PD Vousden KH. Serine metabolism supports the methionine cycle and DNA/RNA methylation through *De novo* ATP synthesis in cancer cells. Mol Cell (2016) 61:210–21. doi: 10.1016/j.molcel.2015.12.014 PMC4728077 26774282

[21] Baek SH Kim KI. Epigenetic control of autophagy: Nuclear events gain more attention. Mol Cell (2017) 65:781–5. doi: 10.1016/j.molcel.2016.12.027 28257699

[22] Shi Y Shen HM Gopalakrishnan V Gordon N. Epigenetic regulation of autophagy beyond the cytoplasm: A review. Front Cell Dev Biol (2021) 9:675599. doi: 10.3389/fcell.2021.675599 34195194 PMC8237754

[23] Pearce EL. Metabolism as a driver of immunity. Nat Rev Immunol (2021) 21:618–9. doi: 10.1038/s41577-021-00601-3 34580450

[24] Wolf AJ Reyes CN Liang W Becker C Shimada K Wheeler ML. Hexokinase is an innate immune receptor for the detection of bacterial peptidoglycan. Cell (2016) 166:624–36. doi: 10.1016/j.cell.2016.05.076 PMC5534359 27374331

[25] O’Sullivan D Kelly B Pearce EL. When hexokinase gets that NAG-ing feeling. Cell Metab (2016) 24:198–200. doi: 10.1016/j.cmet.2016.07.021 27508867 PMC5572107

[26] Ahmad A Aboukameel A Kong D Wang Z Sethi S Chen W. Phosphoglucose isomerase/autocrine motility factor mediates epithelial-mesenchymal transition regulated by miR-200 in breast cancer cells. Cancer Res (2011) 71:3400–9. doi: 10.1158/0008-5472.CAN-10-0965 PMC3085607 21389093

[27] Schultze SM Hemmings BA Niessen M Tschopp O. PI3K/AKT, MAPK and AMPK signalling: Protein kinases in glucose homeostasis. Expert Rev Mol Med (2012) 14:e1. doi: 10.1017/S1462399411002109 22233681

[28] Papa S Choy PM Bubici C. The ERK and JNK pathways in the regulation of metabolic reprogramming. Oncogene (2019) 38:2223–40. doi: 10.1038/s41388-018-0582-8 PMC6398583 30487597

[29] Sharif O Brunner JS Vogel A Schabbauer G. Macrophage rewiring by nutrient associated PI3K dependent pathways. Front Immunol (2019) 10:2002. doi: 10.3389/fimmu.2019.02002 31497027 PMC6712174

[30] Bednarczyk RB Tuli NY Hanly EK Rahoma GB Maniyar R Mittelman A. Macrophage inflammatory factors promote epithelial-mesenchymal transition in breast cancer. Oncotarget (2018) 9:24272–82. doi: 10.18632/oncotarget.24917 PMC5966261 29849939

[31] McCarthy JS Wieseman M Tropea J Kaslow D Abraham D Lustigman S. Onchocerca volvulus glycolytic enzyme fructose-1,6-bisphosphate aldolase as a target for a protective immune response in humans. Infect Immun (2002) 70:851–8. doi: 10.1128/IAI.70.2.851-858.2002 PMC127653 11796620

[32] Trujillo C Blumenthal A Marrero J Rhee KY Schnappinger D Ehrt S. Triosephosphate isomerase is dispensable *in vitro* yet essential for mycobacterium tuberculosis to establish infection. mBio (2014) 5:e00085. doi: 10.1128/mBio.00085-14 24757211 PMC3994511

[33] Wang YT Huang HY Tsai MA Wang PC Jiang BH Chen SC. Phosphoglycerate kinase enhanced immunity of the whole cell of streptococcus agalactiae in tilapia, oreochromis niloticus. Fish Shellfish Immunol (2014) 41:250–9. doi: 10.1016/j.fsi.2014.09.008 25218275

[34] Li Z Zhang H Zhang J Xi L Yang G Wang S. Brucella abortus phosphoglyceromutase and dihydrodipicolinate reductase induce Th1 and Th2-related immune responses. World J Microbiol Biotechnol (2018) 34:22. doi: 10.1007/s11274-017-2405-4 29302824

[35] Ryans K Omosun Y McKeithen DN Simoneaux T Mills CC Bowen N. The immunoregulatory role of alpha enolase in dendritic cell function during chlamydia infection. BMC Immunol (2017) 18:27. doi: 10.1186/s12865-017-0212-1 28525970 PMC5437423

[36] Stone OA El-Brolosy M Wilhelm K Liu X Romao AM Grillo E. Loss of pyruvate kinase M2 limits growth and triggers innate immune signaling in endothelial cells. Nat Commun (2018) 9:4077. doi: 10.1038/s41467-018-06406-8 30301887 PMC6177464

[37] Millet P Vachharajani V McPhail L Yoza B McCall CE. GAPDH binding to TNF-alpha mRNA contributes to posttranscriptional repression in monocytes: A novel mechanism of communication between inflammation and metabolism. J Immunol (2016) 196:2541–51. doi: 10.4049/jimmunol.1501345 PMC4779706 26843329

[38] Min BK Park S Kang HJ Kim DW Ham HJ Ha CM. Pyruvate dehydrogenase kinase is a metabolic checkpoint for polarization of macrophages to the M1 phenotype. Front Immunol (2019) 10:944. doi: 10.3389/fimmu.2019.00944 31134063 PMC6514528

[39] Ge T Yang J Zhou S Wang Y Li Y Tong X. The role of the pentose phosphate pathway in diabetes and cancer. Front Endocrinol (Lausanne) (2020) 11:365. doi: 10.3389/fendo.2020.00365 32582032 PMC7296058

[40] Manoharan I Prasad PD Thangaraju M Manicassamy S. Lactate-dependent regulation of immune responses by dendritic cells and macrophages. Front Immunol (2021) 12:691134. doi: 10.3389/fimmu.2021.691134 34394085 PMC8358770

[41] Ho PC Bihuniak JD Macintyre AN Staron M Liu X Amezquita R. Phosphoenolpyruvate is a metabolic checkpoint of anti-tumor T cell responses. Cell (2015) 162:1217–28. doi: 10.1016/j.cell.2015.08.012 PMC4567953 26321681

[42] Stincone A Prigione A Cramer T Wamelink MM Campbell K Cheung E. The return of metabolism: Biochemistry and physiology of the pentose phosphate pathway. Biol Rev Camb Philos Soc (2015) 90:927–63. doi: 10.1111/brv.12140 PMC4470864 25243985

[43] Choi I Son H Baek JH. Tricarboxylic acid (TCA) cycle intermediates: Regulators of immune responses. Life (Basel) (2021) 11. doi: 10.3390/life11010069 PMC7832849 33477822

[44] Williams NC O’Neill LAJ. A role for the Krebs cycle intermediate citrate in metabolic reprogramming in innate immunity and inflammation. Front Immunol (2018) 9:141. doi: 10.3389/fimmu.2018.00141 29459863 PMC5807345

[45] Kurniawan H Kobayashi T Brenner D. The emerging role of one-carbon metabolism in T cells. Curr Opin Biotechnol (2021) 68:193–201. doi: 10.1016/j.copbio.2020.12.001 33422815

[46] Richter FC Clarke AJ. One carbon (metabolism) to rule T cell identity. Nat Rev Immunol (2021) 21:206. doi: 10.1038/s41577-021-00530-1 33750935

[47] Cruzat V Macedo Rogero M Noel Keane K Curi R Newsholme P. Glutamine: Metabolism and immune function, supplementation and clinical translation. Nutrients (2018) 10. doi: 10.3390/nu10111564 PMC6266414 30360490

[48] Bronte V Zanovello P. Regulation of immune responses by l-arginine metabolism. Nat Rev Immunol (2005) 5:641–54. doi: 10.1038/nri1668 16056256

[49] Schieber M Chandel NS. ROS function in redox signaling and oxidative stress. Curr Biol (2014) 24:R453–462. doi: 10.1016/j.cub.2014.03.034 PMC4055301 24845678

[50] Zhao RZ Jiang S Zhang L Yu ZB. Mitochondrial electron transport chain, ROS generation and uncoupling (Review). Int J Mol Med (2019) 44:3–15. doi: 10.3892/ijmm.2019.4188 31115493 PMC6559295

[51] Canto C Menzies KJ Auwerx J. NAD(+) metabolism and the control of energy homeostasis: A balancing act between mitochondria and the nucleus. Cell Metab (2015) 22:31–53. doi: 10.1016/j.cmet.2015.05.023 26118927 PMC4487780

[52] Chenault HK Whitesides GM. Lactate dehydrogenase-catalyzed regenertion of NAD from NADH for use in enzyme-catalyzed synthesis. Biooganic Chem (1989) 17:400–9. doi: 10.1016/0045-2068(89)90041-2

[53] Vermot A Petit-Hartlein I Smith SME Fieschi F. NADPH oxidases (NOX): An overview from discovery, molecular mechanisms to physiology and pathology. Antioxidants (Basel) (2021) 10. doi: 10.3390/antiox10060890 PMC8228183 34205998

[54] Bedard K Krause KH. The NOX family of ROS-generating NADPH oxidases: physiology and pathophysiology. Physiol Rev (2007) 87:245–313. doi: 10.1152/physrev.00044.2005 17237347

[55] Cross AR Segal AW. The NADPH oxidase of professional phagocytes–prototype of the NOX electron transport chain systems. Biochim Biophys Acta (2004) 1657:1–22. doi: 10.1016/j.bbabio.2004.03.008 PMC2636547 15238208

[56] Tannahill GM Curtis AM Adamik J Palsson-McDermott EM McGettrick AF Goel G. Succinate is an inflammatory signal that induces IL-1beta through HIF-1alpha. Nature (2013) 496:238–42. doi: 10.1038/nature11986 PMC4031686 23535595

[57] Hadrava Vanova K Kraus M Neuzil J Rohlena J. Mitochondrial complex II and reactive oxygen species in disease and therapy. Redox Rep (2020) 25:26–32. doi: 10.1080/13510002.2020.1752002 32290794 PMC7178880

[58] Speijer D. Can all major ROS forming sites of the respiratory chain be activated by high FADH2/NADH ratios?: ancient evolutionary constraints determine mitochondrial ROS formation. Bioessays (2019) 41:e1800180. doi: 10.1002/bies.201800180 30512221

[59] Hall CJ Boyle RH Astin JW Flores MV Oehlers SH Sanderson LE. Immunoresponsive gene 1 augments bactericidal activity of macrophage-lineage cells by regulating beta-oxidation-dependent mitochondrial ROS production. Cell Metab (2013) 18:265–78. doi: 10.1016/j.cmet.2013.06.018 23931757

[60] Jaiswal AK Yadav J Makhija S Mazumder S Mitra AK Suryawanshi A. Irg1/itaconate metabolic pathway is a crucial determinant of dendritic cells immune-priming function and contributes to resolute allergen-induced airway inflammation. Mucosal Immunol (2022) 15:301–13. doi: 10.1038/s41385-021-00462-y PMC8866123 34671116

[61] Sasikaran J Ziemski M Zadora PK Fleig A Berg IA. Bacterial itaconate degradation promotes pathogenicity. Nat Chem Biol (2014) 10:371–7. doi: 10.1038/nchembio.1482 24657929

[62] Michelucci A Cordes T Ghelfi J Pailot A Reiling N Goldmann O. Immune-responsive gene 1 protein links metabolism to immunity by catalyzing itaconic acid production. Proc Natl Acad Sci U.S.A. (2013) 110:7820–5. 10.1073/pnas.1218599110 PMC3651434 23610393

[63] O’Neill LAJ Artyomov MN. Itaconate: the poster child of metabolic reprogramming in macrophage function. Nat Rev Immunol (2019) 19:273–81. doi: 10.1038/s41577-019-0128-5 30705422

[64] Canton M Sanchez-Rodriguez R Spera I Venegas FC Favia M Viola A. Reactive oxygen species in macrophages: Sources and targets. Front Immunol (2021) 12:734229. doi: 10.3389/fimmu.2021.734229 34659222 PMC8515906

[65] Tan HY Wang N Li S Hong M Wang X Feng Y. The reactive oxygen species in macrophage polarization: Reflecting its dual role in progression and treatment of human diseases. Oxid Med Cell Longev (2016) 2016:2795090. doi: 10.1155/2016/2795090 27143992 PMC4837277

[66] Radi R. Oxygen radicals, nitric oxide, and peroxynitrite: Redox pathways in molecular medicine. Proc Natl Acad Sci U.S.A. (2018) 115:5839–48. 10.1073/pnas.1804932115 PMC6003358 29802228

[67] Weinberg JB. Nitric oxide production and nitric oxide synthase type 2 expression by human mononuclear phagocytes: A review. Mol Med (1998) 4:557–91. doi: 10.1007/BF03401758 PMC2230318 9848075

[68] Mori M Gotoh T. Regulation of nitric oxide production by arginine metabolic enzymes. Biochem Biophys Res Commun (2000) 275:715–9. doi: 10.1006/bbrc.2000.3169 10973788

[69] Herb M Schramm M. Functions of ROS in macrophages and antimicrobial immunity. Antioxidants (Basel) (2021). doi: 10.3390/antiox10020313 PMC7923022 33669824

[70] Ding AH Nathan CF Stuehr DJ. Release of reactive nitrogen intermediates and reactive oxygen intermediates from mouse peritoneal macrophages. Comparison Activating Cytokines Evidence Independent Production J Immunol (1988) 141:2407–12. 3139757

[71] Drapier JC Wietzerbin J Hibbs JB Jr. Interferon-gamma and tumor necrosis factor induce the l-arginine-dependent cytotoxic effector mechanism in murine macrophages. Eur J Immunol (1988) 18:1587–92. doi: 10.1002/eji.1830181018 3142779

[72] Lyons CR Orloff GJ Cunningham JM. Molecular cloning and functional expression of an inducible nitric oxide synthase from a murine macrophage cell line. J Biol Chem (1992) 267:6370–4. doi: 10.1016/S0021-9258(18)42704-4 1372907

[73] Zhong J Scholz T Yau ACY Guerard S Huffmeier U Burkhardt H. Mannan-induced Nos2 in macrophages enhances IL-17-driven psoriatic arthritis by innate lymphocytes. Sci Adv (2018) 4:eaas9864. doi: 10.1126/sciadv.aas9864 29774240 PMC5955621

[74] Baran CP Zeigler MM Tridandapani S Marsh CB. The role of ROS and RNS in regulating life and death of blood monocytes. Curr Pharm Des (2004) 10:855–66. doi: 10.2174/1381612043452866 15032689

[75] Iovine NM Pursnani S Voldman A Wasserman G Blaser MJ Weinrauch Y. Reactive nitrogen species contribute to innate host defense against campylobacter jejuni. Infect Immun (2008) 76:986–93. doi: 10.1128/IAI.01063-07 PMC2258852 18174337

[76] Canton J Khezri R Glogauer M Grinstein S. Contrasting phagosome pH regulation and maturation in human M1 and M2 macrophages. Mol Biol Cell (2014) 25:3330–41. doi: 10.1091/mbc.e14-05-0967 PMC4214780 25165138

[77] Bailey JD Diotallevi M Nicol T McNeill E Shaw A Chuaiphichai S. Nitric oxide modulates metabolic remodeling in inflammatory macrophages through TCA cycle regulation and itaconate accumulation. Cell Rep (2019) 28:218–230 e217. doi: 10.1016/j.celrep.2019.06.018 31269442 PMC6616861

[78] Nair S Huynh JP Lampropoulou V Loginicheva E Esaulova E Gounder AP. Irg1 expression in myeloid cells prevents immunopathology during m. tuberculosis infection. J Exp Med (2018) 215:1035–45. doi: 10.1084/jem.20180118 PMC5881474 29511063

[79] Paiva CN Bozza MT. Are reactive oxygen species always detrimental to pathogens? Antioxid Redox Signal (2014) 20:1000–37. doi: 10.1089/ars.2013.5447 PMC3924804 23992156

[80] Palmieri EM Gonzalez-Cotto M Baseler WA Davies LC Ghesquiere B Maio N. Nitric oxide orchestrates metabolic rewiring in M1 macrophages by targeting aconitase 2 and pyruvate dehydrogenase. Nat Commun (2020) 11:698. doi: 10.1038/s41467-020-14433-7 32019928 PMC7000728

[81] Shi L Jiang Q Bushkin Y Subbian S Tyagi S. Biphasic dynamics of macrophage immunometabolism during mycobacterium tuberculosis infection. mBio (2019) 10. doi: 10.1128/mBio.02550-18 PMC6437057 30914513

[82] Howard NC Khader SA. Immunometabolism during mycobacterium tuberculosis infection. Trends Microbiol (2020) 28:832–50. doi: 10.1016/j.tim.2020.04.010 PMC7494650 32409147

[83] Park JH Shim D Kim KES Lee W Shin SJ. Understanding metabolic regulation between host and pathogens: New opportunities for the development of improved therapeutic strategies against mycobacterium tuberculosis infection. Front Cell Infect Microbiol (2021) 11:635335. doi: 10.3389/fcimb.2021.635335 33796480 PMC8007978

[84] Shi L Salamon H Eugenin EA Pine R Cooper A Gennaro ML. Infection with mycobacterium tuberculosis induces the warburg effect in mouse lungs. Sci Rep (2015) 5:18176. doi: 10.1038/srep18176 26658723 PMC4674750

[85] Diskin C Palsson-McDermott EM. Metabolic modulation in macrophage effector function. Front Immunol (2018) 9:270. doi: 10.3389/fimmu.2018.00270 29520272 PMC5827535

[86] Sowers ML Tang H Singh VK Khan A Mishra A Restrepo BI. Multi-OMICs analysis reveals metabolic and epigenetic changes associated with macrophage polarization. J Biol Chem (2022) 298:102418. doi: 10.1016/j.jbc.2022.102418 36030823 PMC9525912

[87] Cox DJ Coleman AM Gogan KM Phelan JJ Dunne PJ Basdeo SA. Inhibiting histone deacetylases in human macrophages promotes glycolysis, IL-1beta, and T helper cell responses to mycobacterium tuberculosis. Front Immunol (2020) 11:1609. doi: 10.3389/fimmu.2020.01609 32793237 PMC7390906

[88] Cumming BM Addicott KW Adamson JH Steyn AJ. Mycobacterium tuberculosis induces decelerated bioenergetic metabolism in human macrophages. Elife (2018) 7. doi: 10.7554/eLife.39169 PMC6286123 30444490

[89] Mishra A Singh VK Jagannath C Subbian S Restrepo BI Gauduin MC. Human macrophages exhibit GM-CSF dependent restriction of mycobacterium tuberculosis infection *via* regulating their self-survival, differentiation and metabolism. Front Immunol (2022) 13:859116. doi: 10.3389/fimmu.2022.859116 35634283 PMC9134823

[90] Phelan JJ McQuaid K Kenny C Gogan KM Cox DJ Basdeo SA. Desferrioxamine supports metabolic function in primary human macrophages infected with mycobacterium tuberculosis. Front Immunol (2020) 11:836. doi: 10.3389/fimmu.2020.00836 32477344 PMC7237728

[91] van Doorn CLR Steenbergen SAM Walburg KV Ottenhoff THM. Pharmacological poly (ADP-ribose) polymerase inhibitors decrease mycobacterium tuberculosis survival in human macrophages. Front Immunol (2021) 12:712021. doi: 10.3389/fimmu.2021.712021 34899683 PMC8662539

[92] Pu W Zhao C Wazir J Su Z Niu M Song S. Comparative transcriptomic analysis of THP-1-derived macrophages infected with mycobacterium tuberculosis H37Rv, H37Ra and BCG. J Cell Mol Med (2021) 25:10504–20. doi: 10.1111/jcmm.16980 PMC8581329 34632719

[93] Gleeson LE Sheedy FJ Palsson-McDermott EM Triglia D O’Leary SM O’Sullivan MP. Cutting edge: Mycobacterium tuberculosis induces aerobic glycolysis in human alveolar macrophages that is required for control of intracellular bacillary replication. J Immunol (2016) 196:2444–9. doi: 10.4049/jimmunol.1501612 26873991

[94] Cox DJ Phelan JJ Mitermite M Murphy DM Leisching G Thong L. Lactate alters metabolism in human macrophages and improves their ability to kill mycobacterium tuberculosis. Front Immunol (2021) 12:663695. doi: 10.3389/fimmu.2021.663695 34691015 PMC8526932

[95] Cahill C Cox DJ O’Connell F Basdeo SA Gogan KM O’Maoldomhnaigh C. The effect of tuberculosis antimicrobials on the immunometabolic profiles of primary human macrophages stimulated with mycobacterium tuberculosis. Int J Mol Sci (2021) 22. doi: 10.3390/ijms222212189 PMC8624646 34830070

[96] Roy S Schmeier S Kaczkowski B Arner E Alam T Ozturk M. Transcriptional landscape of mycobacterium tuberculosis infection in macrophages. Sci Rep (2018) 8:6758. doi: 10.1038/s41598-018-24509-6 29712924 PMC5928056

[97] Martinez J Verbist K Wang R Green DR. The relationship between metabolism and the autophagy machinery during the innate immune response. Cell Metab (2013) 17:895–900. doi: 10.1016/j.cmet.2013.05.012 23747248 PMC3696504

[98] Galluzzi L Pietrocola F Levine B Kroemer G. Metabolic control of autophagy. Cell (2014) 159:1263–76. doi: 10.1016/j.cell.2014.11.006 PMC4500936 25480292

[99] Marino G Pietrocola F Eisenberg T Kong Y Malik SA Andryushkova A. Regulation of autophagy by cytosolic acetyl-coenzyme a. Mol Cell (2014) 53:710–25. doi: 10.1016/j.molcel.2014.01.016 24560926

[100] Ye J Kumanova M Hart LS Sloane K Zhang H De Panis DN. The GCN2-ATF4 pathway is critical for tumour cell survival and proliferation in response to nutrient deprivation. EMBO J (2010) 29:2082–96. doi: 10.1038/emboj.2010.81 PMC2892366 20473272

[101] Roberts DJ Tan-Sah VP Ding EY Smith JM Miyamoto S. Hexokinase-II positively regulates glucose starvation-induced autophagy through TORC1 inhibition. Mol Cell (2014) 53:521–33. doi: 10.1016/j.molcel.2013.12.019 PMC3943874 24462113

[102] Kundu M. Too sweet for autophagy: Hexokinase inhibition of mTORC1 activates autophagy. Mol Cell (2014) 53:517–8. doi: 10.1016/j.molcel.2014.02.009 PMC4106709 24560270

[103] Moonira T Chachra SS Ford BE Marin S Alshawi A Adam-Primus NS. Metformin lowers glucose 6-phosphate in hepatocytes by activation of glycolysis downstream of glucose phosphorylation. J Biol Chem (2020) 295:3330–46. doi: 10.1074/jbc.RA120.012533 PMC7062158 31974165

[104] De Santi M Baldelli G Diotallevi A Galluzzi L Schiavano GF Brandi G. Metformin prevents cell tumorigenesis through autophagy-related cell death. Sci Rep (2019) 9:66. doi: 10.1038/s41598-018-37247-6 30635619 PMC6329809

[105] Cruz CM Rinna A Forman HJ Ventura AL Persechini PM Ojcius DM. ATP activates a reactive oxygen species-dependent oxidative stress response and secretion of proinflammatory cytokines in macrophages. J Biol Chem (2007) 282:2871–9. doi: 10.1074/jbc.M608083200 PMC2693903 17132626

[106] Egan D Kim J Shaw RJ Guan KL. The autophagy initiating kinase ULK1 is regulated *via* opposing phosphorylation by AMPK and mTOR. Autophagy (2011) 7:643–4. doi: 10.4161/auto.7.6.15123 PMC3359466 21460621

[107] Kim J Kundu M Viollet B Guan KL. AMPK and mTOR regulate autophagy through direct phosphorylation of Ulk1. Nat Cell Biol (2011) 13:132–41. doi: 10.1038/ncb2152 PMC3987946 21258367

[108] Hardie DG. Minireview: The AMP-activated protein kinase cascade: The key sensor of cellular energy status. Endocrinology (2003) 144:5179–83. doi: 10.1210/en.2003-0982 12960015

[109] Gwinn DM Shackelford DB Egan DF Mihaylova MM Mery A Vasquez DS. AMPK phosphorylation of raptor mediates a metabolic checkpoint. Mol Cell (2008) 30:214–26. doi: 10.1016/j.molcel.2008.03.003 PMC2674027 18439900

[110] Inoki K Li Y Xu T Guan KL. Rheb GTPase is a direct target of TSC2 GAP activity and regulates mTOR signaling. Genes Dev (2003) 17:1829–34. doi: 10.1101/gad.1110003 PMC196227 12869586

[111] Yorimitsu T Zaman S Broach JR Klionsky DJ. Protein kinase a and Sch9 cooperatively regulate induction of autophagy in saccharomyces cerevisiae. Mol Biol Cell (2007) 18:4180–9. doi: 10.1091/mbc.e07-05-0485 PMC1995722 17699586

[112] Stephan JS Yeh YY Ramachandran V Deminoff SJ Herman PK. The tor and PKA signaling pathways independently target the Atg1/Atg13 protein kinase complex to control autophagy. Proc Natl Acad Sci U.S.A. (2009) 106:17049–54. 10.1073/pnas.0903316106 PMC2761351 19805182

[113] Menon MB Dhamija S. Beclin 1 phosphorylation - at the center of autophagy regulation. Front Cell Dev Biol (2018) 6:137. doi: 10.3389/fcell.2018.00137 30370269 PMC6194997

[114] Wang RC Wei Y An Z Zou Z Xiao G Bhagat G. Akt-mediated regulation of autophagy and tumorigenesis through beclin 1 phosphorylation. Science (2012) 338:956–9. doi: 10.1126/science.1225967 PMC3507442 23112296

[115] Qian X Li X Cai Q Zhang C Yu Q Jiang Y. Phosphoglycerate kinase 1 phosphorylates Beclin1 to induce autophagy. Mol Cell (2017) 65:917–931 e916. doi: 10.1016/j.molcel.2017.01.027 28238651 PMC5389741

[116] Huang X Liu G Guo J Su Z. The PI3K/AKT pathway in obesity and type 2 diabetes. Int J Biol Sci (2018) 14:1483–96. doi: 10.7150/ijbs.27173 PMC6158718 30263000

[117] Hoxhaj G Manning BD. The PI3K-AKT network at the interface of oncogenic signalling and cancer metabolism. Nat Rev Cancer (2020) 20:74–88. doi: 10.1038/s41568-019-0216-7 31686003 PMC7314312

[118] Vergadi E Ieronymaki E Lyroni K Vaporidi K Tsatsanis C. Akt signaling pathway in macrophage activation and M1/M2 polarization. J Immunol (2017) 198:1006–14. doi: 10.4049/jimmunol.1601515 28115590

[119] Zubova SG Suvorova II Karpenko MN. Macrophage and microglia polarization: Focus on autophagy-dependent reprogramming. Front Biosci (Schol Ed) (2022) 14:3. doi: 10.31083/j.fbs1401003 35320914

[120] Yu X Li L Xia L Feng X Chen F Cao S. Impact of metformin on the risk and treatment outcomes of tuberculosis in diabetics: a systematic review. BMC Infect Dis (2019) 19:859. doi: 10.1186/s12879-019-4548-4 31623569 PMC6796338

[121] Collins SL Oh MH Sun IH Chan-Li Y Zhao L Powell JD. mTORC1 signaling regulates proinflammatory macrophage function and metabolism. J Immunol (2021) 207:913–22. doi: 10.4049/jimmunol.2100230 34290107

[122] Mercalli A Calavita I Dugnani E Citro A Cantarelli E Nano R. Rapamycin unbalances the polarization of human macrophages to M1. Immunology (2013) 140:179–90. doi: 10.1111/imm.12126 PMC3784164 23710834

[123] Moruno-Manchon JF Perez-Jimenez E Knecht E. Glucose induces autophagy under starvation conditions by a p38 MAPK-dependent pathway. Biochem J (2013) 449:497–506. doi: 10.1042/BJ20121122 23116132

[124] Zhang D Tang Z Huang H Zhou G Cui C Weng Y. Metabolic regulation of gene expression by histone lactylation. Nature (2019) 574:575–80. doi: 10.1038/s41586-019-1678-1 PMC6818755 31645732

[125] Noe JT Rendon BE Geller AE Conroy LR Morrissey SM Young LEA. Lactate supports a metabolic-epigenetic link in macrophage polarization. Sci Adv (2021) 7:eabi8602. doi: 10.1126/sciadv.abi8602 34767443 PMC8589316

[126] Guarente L. The logic linking protein acetylation and metabolism. Cell Metab (2011) 14:151–3. doi: 10.1016/j.cmet.2011.07.007 21803285

[127] Anderson KA Hirschey MD. Mitochondrial protein acetylation regulates metabolism. Essays Biochem (2012) 52:23–35. doi: 10.1042/bse0520023 22708561 PMC3872051

[128] Mukherjee S Hao YH Orth K. A newly discovered post-translational modification–the acetylation of serine and threonine residues. Trends Biochem Sci (2007) 32:210–6. doi: 10.1016/j.tibs.2007.03.007 17412595

[129] Shi L Tu BP. Acetyl-CoA and the regulation of metabolism: Mechanisms and consequences. Curr Opin Cell Biol (2015) 33:125–31. doi: 10.1016/j.ceb.2015.02.003 PMC4380630 25703630

[130] Watson JA Fang M Lowenstein JM. Tricarballylate and hydroxycitrate: Substrate and inhibitor of ATP: Citrate oxaloacetate lyase. Arch Biochem Biophys (1969) 135:209–17. doi: 10.1016/0003-9861(69)90532-3 5362924

[131] Huang W Wang Z Lei QY. Acetylation control of metabolic enzymes in cancer: an updated version. Acta Biochim Biophys Sin (Shanghai) (2014) 46:204–13. doi: 10.1093/abbs/gmt154 24480802

[132] Kwon OS Han MJ Cha HJ. Suppression of SIRT2 and altered acetylation status of human pluripotent stem cells: Possible link to metabolic switch during reprogramming. BMB Rep (2017) 50:435–6. doi: 10.5483/BMBRep.2017.50.9.119 PMC5625689 28683850

[133] Lv L Li D Zhao D Lin R Chu Y Zhang H. Acetylation targets the M2 isoform of pyruvate kinase for degradation through chaperone-mediated autophagy and promotes tumor growth. Mol Cell (2011) 42:719–30. doi: 10.1016/j.molcel.2011.04.025 PMC4879880 21700219

[134] Park SH Ozden O Liu G Song HY Zhu Y Yan Y. SIRT2-mediated deacetylation and tetramerization of pyruvate kinase directs glycolysis and tumor growth. Cancer Res (2016) 76:3802–12. doi: 10.1158/0008-5472.CAN-15-2498 PMC4930699 27197174

[135] Bhardwaj A Das S. SIRT6 deacetylates PKM2 to suppress its nuclear localization and oncogenic functions. Proc Natl Acad Sci U.S.A. (2016) 113:E538–547. doi: 10.1073/pnas.1520045113 PMC4747762 26787900

[136] Peeters K Van Leemputte F Fischer B Bonini BM Quezada H Tsytlonok M. Fructose-1,6-bisphosphate couples glycolytic flux to activation of ras. Nat Commun (2017) 8:922. doi: 10.1038/s41467-017-01019-z 29030545 PMC5640605

[137] Tan VP Miyamoto S. HK2/hexokinase-II integrates glycolysis and autophagy to confer cellular protection. Autophagy (2015) 11:963–4. doi: 10.1080/15548627.2015.1042195 PMC4502787 26075878

[138] Wang F Wang K Xu W Zhao S Ye D Wang Y. SIRT5 desuccinylates and activates pyruvate kinase M2 to block macrophage IL-1beta production and to prevent DSS-induced colitis in mice. Cell Rep (2017) 19:2331–44. doi: 10.1016/j.celrep.2017.05.065 28614718

[139] Yucel N Wang YX Mai T Porpiglia E Lund PJ Markov G. Glucose metabolism drives histone acetylation landscape transitions that dictate muscle stem cell function. Cell Rep (2019) 27:3939–3955 e3936. doi: 10.1016/j.celrep.2019.05.092 31242425 PMC6788807

[140] Zhao S Torres A Henry RA Trefely S Wallace M Lee JV. ATP-citrate lyase controls a glucose-to-Acetate metabolic switch. Cell Rep (2016) 17:1037–52. doi: 10.1016/j.celrep.2016.09.069 PMC5175409 27760311

[141] Deb DK Chen Y Sun J Wang Y Li YC. ATP-citrate lyase is essential for high glucose-induced histone hyperacetylation and fibrogenic gene upregulation in mesangial cells. Am J Physiol Renal Physiol (2017) 313:F423–9. doi: 10.1152/ajprenal.00029.2017 28490526

[142] Wellen KE Hatzivassiliou G Sachdeva UM Bui TV Cross JR Thompson CB. ATP-citrate lyase links cellular metabolism to histone acetylation. Science (2009) 324:1076–80. doi: 10.1126/science.1164097 PMC2746744 19461003

[143] Ivashkiv LB. Epigenetic regulation of macrophage polarization and function. Trends Immunol (2013) 34:216–23. doi: 10.1016/j.it.2012.11.001 PMC3647003 23218730

[144] Torres A Makowski L Wellen KE. Immunometabolism: Metabolism fine-tunes macrophage activation. Elife (2016) 5. doi: 10.7554/eLife.14354 PMC4769164 26894957

[145] Dong Z Li R Xu L Xin K Xu Y Shi H. Histone hyperacetylation mediates enhanced IL-1beta production in LPS/IFN-gamma-stimulated macrophages. Immunology (2020) 160:183–97. doi: 10.1111/imm.13183 PMC7218666 32061096

[146] Dominguez M Brune B Namgaladze D. Exploring the role of ATP-citrate lyase in the immune system. Front Immunol (2021) 12:632526. doi: 10.3389/fimmu.2021.632526 33679780 PMC7930476

[147] Lauterbach MA Hanke JE Serefidou M Mangan MSJ Kolbe CC Hess T. Toll-like receptor signaling rewires macrophage metabolism and promotes histone acetylation *via* ATP-citrate lyase. Immunity (2019) 51:997–1011 e1017. doi: 10.1016/j.immuni.2019.11.009 31851905

[148] denDekker AD Davis FM Joshi AD Wolf SJ Allen R Lipinski J. TNF-alpha regulates diabetic macrophage function through the histone acetyltransferase MOF. JCI Insight (2020) 5. doi: 10.1172/jci.insight.132306 PMC7141388 32069267

[149] Kelly B O’Neill LA. Metabolic reprogramming in macrophages and dendritic cells in innate immunity. Cell Res (2015) 25:771–84. doi: 10.1038/cr.2015.68 PMC4493277 26045163

[150] Vlad ML Manea SA Lazar AG Raicu M Muresian H Simionescu M. Histone acetyltransferase-dependent pathways mediate upregulation of NADPH oxidase 5 in human macrophages under inflammatory conditions: A potential mechanism of reactive oxygen species overproduction in atherosclerosis. Oxid Med Cell Longev (2019) 2019 3201062. doi: 10.1155/2019/3201062 31565149 PMC6745143

[151] Bories GFP Leitinger N. Macrophage metabolism in atherosclerosis. FEBS Lett (2017) 591:3042–60. doi: 10.1002/1873-3468.12786 28796886

[152] Baardman J Verberk SGS van der Velden S Gijbels MJJ van Roomen C Sluimer JC. Macrophage ATP citrate lyase deficiency stabilizes atherosclerotic plaques. Nat Commun (2020) 11:6296. doi: 10.1038/s41467-020-20141-z 33293558 PMC7722882

[153] Infantino V Convertini P Cucci L Panaro MA Di Noia MA Calvello R. The mitochondrial citrate carrier: A new player in inflammation. Biochem J (2011) 438:433–6. doi: 10.1042/BJ20111275 21787310

[154] Infantino V Iacobazzi V Palmieri F Menga A. ATP-citrate lyase is essential for macrophage inflammatory response. Biochem Biophys Res Commun (2013) 440:105–11. doi: 10.1016/j.bbrc.2013.09.037 24051091

[155] Namgaladze D Zukunft S Schnutgen F Kurrle N Fleming I Fuhrmann D. Polarization of human macrophages by interleukin-4 does not require ATP-citrate lyase. Front Immunol (2018) 9:2858. doi: 10.3389/fimmu.2018.02858 30568658 PMC6290342

[156] Fullgrabe J Lynch-Day MA Heldring N Li W Struijk RB Ma Q. The histone H4 lysine 16 acetyltransferase hMOF regulates the outcome of autophagy. Nature (2013) 500:468–71. doi: 10.1038/nature12313 PMC4006103 23863932

[157] Hamaidi I Kim S. Sirtuins are crucial regulators of T cell metabolism and functions. Exp Mol Med (2022) 54:207–15. doi: 10.1038/s12276-022-00739-7 PMC8979958 35296782

[158] Vachharajani VT Liu T Wang X Hoth JJ Yoza BK McCall CE. Sirtuins link inflammation and metabolism. J Immunol Res (2016) 2016:8167273. doi: 10.1155/2016/8167273 26904696 PMC4745579

[159] Warren JL MacIver NJ. Regulation of adaptive immune cells by sirtuins. Front Endocrinol (Lausanne) (2019) 10:466. doi: 10.3389/fendo.2019.00466 31354630 PMC6637536

[160] Anderson KA Madsen AS Olsen CA Hirschey MD. Metabolic control by sirtuins and other enzymes that sense NAD(+), NADH, or their ratio. Biochim Biophys Acta Bioenerg (2017) 1858:991–8. doi: 10.1016/j.bbabio.2017.09.005 PMC5648639 28947253

[161] Aguilar-Arnal L Ranjit S Stringari C Orozco-Solis R Gratton E Sassone-Corsi P. Spatial dynamics of SIRT1 and the subnuclear distribution of NADH species. Proc Natl Acad Sci U.S.A. (2016) 113:12715–20. 10.1073/pnas.1609227113 PMC5111721 27791113

[162] Liang F Kume S Koya D. SIRT1 and insulin resistance. Nat Rev Endocrinol (2009) 5:367–73. doi: 10.1038/nrendo.2009.101 19455179

[163] Moynihan KA Grimm AA Plueger MM Bernal-Mizrachi E Ford E Cras-Meneur C. Increased dosage of mammalian Sir2 in pancreatic beta cells enhances glucose-stimulated insulin secretion in mice. Cell Metab (2005) 2:105–17. doi: 10.1016/j.cmet.2005.07.001 16098828

[164] Bordone L Motta MC Picard F Robinson A Jhala US Apfeld J. Sirt1 regulates insulin secretion by repressing UCP2 in pancreatic beta cells. PloS Biol (2006) 4:e31. 16366736 10.1371/journal.pbio.0040031 PMC1318478

[165] Jia Y Zheng Z Wang Y Zhou Q Cai W Jia W. SIRT1 is a regulator in high glucose-induced inflammatory response in RAW264.7 cells. PloS One (2015) 10:e0120849. 25793995 10.1371/journal.pone.0120849 PMC4368832

[166] Civitarese AE Carling S Heilbronn LK Hulver MH Ukropcova B Deutsch WA. Calorie restriction increases muscle mitochondrial biogenesis in healthy humans. PloS Med (2007) 4:e76. doi: 10.1371/journal.pmed.0040076 17341128 PMC1808482

[167] Bordone L Guarente L. Calorie restriction, SIRT1 and metabolism: Understanding longevity. Nat Rev Mol Cell Biol (2005) 6:298–305. doi: 10.1038/nrm1616 15768047

[168] Latifkar A Ling L Hingorani A Johansen E Clement A Zhang X. Loss of sirtuin 1 alters the secretome of breast cancer cells by impairing lysosomal integrity. Dev Cell (2019) 49:393–408 e397. doi: 10.1016/j.devcel.2019.03.011 30982660 PMC6519475

[169] Di Malta C Cinque L Settembre C. Transcriptional regulation of autophagy: Mechanisms and diseases. Front Cell Dev Biol (2019) 7:114. doi: 10.3389/fcell.2019.00114 31312633 PMC6614182

[170] Lee IH Cao L Mostoslavsky R Lombard DB Liu J Bruns NE. A role for the NAD-dependent deacetylase Sirt1 in the regulation of autophagy. Proc Natl Acad Sci U.S.A. (2008) 105:3374–9. 10.1073/pnas.0712145105 PMC2265142 18296641

[171] Price NL Gomes AP Ling AJ Duarte FV Martin-Montalvo A North BJ. SIRT1 is required for AMPK activation and the beneficial effects of resveratrol on mitochondrial function. Cell Metab (2012) 15:675–90. doi: 10.1016/j.cmet.2012.04.003 PMC3545644 22560220

[172] Ghosh HS McBurney M Robbins PD. SIRT1 negatively regulates the mammalian target of rapamycin. PloS One (2010) 5:e9199. doi: 10.1371/journal.pone.0009199 20169165 PMC2821410

[173] Cheng CY Gutierrez NM Marzuki MB Lu X Foreman TW Paleja B. Host sirtuin 1 regulates mycobacterial immunopathogenesis and represents a therapeutic target against tuberculosis. Sci Immunol (2017) 2. doi: 10.1126/sciimmunol.aaj1789 PMC5505666 28707004

[174] Yang H Hu J Chen YJ Ge B. Role of Sirt1 in innate immune mechanisms against mycobacterium tuberculosis *via* the inhibition of TAK1 activation. Arch Biochem Biophys (2019) 667:49–58. doi: 10.1016/j.abb.2019.04.006 31029687

[175] Yang H Chen J Chen Y Jiang Y Ge B Hong L. Sirtuin inhibits m. tuberculosis -induced apoptosis in macrophage through glycogen synthase kinase-3beta. Arch Biochem Biophys (2020) 694:108612. doi: 10.1016/j.abb.2020.108612 33007281

[176] Yang H Chen J Chen Y Jiang Y Ge B Hong L. Sirt1 activation negatively regulates overt apoptosis in mtb-infected macrophage through bax. Int Immunopharmacol (2021) 91:107283. doi: 10.1016/j.intimp.2020.107283 33373810

[177] Wang J Kim TH Ahn MY Lee J Jung JH Choi WS. Sirtinol, a class III HDAC inhibitor, induces apoptotic and autophagic cell death in MCF-7 human breast cancer cells. Int J Oncol (2012) 41:1101–9. doi: 10.3892/ijo.2012.1534 22751989

[178] Ng F Tang BL. Sirtuins’ modulation of autophagy. J Cell Physiol (2013) 228:2262–70. doi: 10.1002/jcp.24399 23696314

[179] Klein MA Denu JM. Biological and catalytic functions of sirtuin 6 as targets for small-molecule modulators. J Biol Chem (2020) 295:11021–41. doi: 10.1074/jbc.REV120.011438 PMC7415977 32518153

[180] Bresque M Cal K Perez-Torrado V Colman L Rodriguez-Duarte J Vilaseca C. SIRT6 stabilization and cytoplasmic localization in macrophages regulates acute and chronic inflammation in mice. J Biol Chem (2022) 298:101711. doi: 10.1016/j.jbc.2022.101711 35150745 PMC8913316

[181] Dantoft W Robertson KA Watkins WJ Strobl B Ghazal P. Metabolic regulators nampt and Sirt6 serially participate in the macrophage interferon antiviral cascade. Front Microbiol (2019) 10:355. doi: 10.3389/fmicb.2019.00355 30886604 PMC6409323

[182] Praveen Prakhar BB Mukherjee T Kolthur-Seetharam U Sundaresan NR Rajmani RS Balaji KN. G9a and Sirtuin6 epigenetically modulate host cholesterol accumulation to facilitate mycobacterial survival. bioRxiv (2021). doi: 10.1101/2021.02.27.433201 PMC10621959 37871034

[183] Blank MF Chen S Poetz F Schnolzer M Voit R Grummt I. SIRT7-dependent deacetylation of CDK9 activates RNA polymerase II transcription. Nucleic Acids Res (2017) 45:2675–86. doi: 10.1093/nar/gkx053 PMC5389538 28426094

[184] Blank MF Grummt I. The seven faces of SIRT7. Transcription (2017) 8:67–74. doi: 10.1080/21541264.2016.1276658 28067587 PMC5423475

[185] Simonet NG Thackray JK Vazquez BN Ianni A Espinosa-Alcantud M Morales-Sanfrutos J. SirT7 auto-ADP-ribosylation regulates glucose starvation response through mH2A1. Sci Adv (2020) 6:eaaz2590. doi: 10.1126/sciadv.aaz2590 32832656 PMC7439345

[186] Hamaidi I Zhang L Kim N Wang MH Iclozan C Fang B. Sirt2 inhibition enhances metabolic fitness and effector functions of tumor-reactive T cells. Cell Metab (2020) 32:420–436 e412. doi: 10.1016/j.cmet.2020.07.008 32768387 PMC7484212

[187] Roychowdhury S Gandhirajan A Kibler C Wang X Vachharajani V. Sirtuin 2 dysregulates autophagy in high-Fat-Exposed immune-tolerant macrophages. Cells (2021) 10. doi: 10.3390/cells10040731 PMC8066127 33810233

[188] Cardoso F Castro F Moreira-Teixeira L Sousa J Torrado E Silvestre R. Myeloid sirtuin 2 expression does not impact long-term mycobacterium tuberculosis control. PloS One (2015) 10:e0131904. doi: 10.1371/journal.pone.0131904 26135889 PMC4489762

[189] Kurundkar D Kurundkar AR Bone NB Becker EJ Jr. Liu W Chacko B. SIRT3 diminishes inflammation and mitigates endotoxin-induced acute lung injury. JCI Insight (2019) 4. doi: 10.1172/jci.insight.120722 PMC6485358 30626741

[190] Smulan LJ Martinez N Kiritsy MC Kativhu C Cavallo K Sassetti CM. Sirtuin 3 downregulation in mycobacterium tuberculosis-infected macrophages reprograms mitochondrial metabolism and promotes cell death. mBio (2021) 12. doi: 10.1128/mBio.03140-20 PMC7858060 33531400

[191] Kim TS Jin YB Kim YS Kim S Kim JK Lee HM. SIRT3 promotes antimycobacterial defenses by coordinating mitochondrial and autophagic functions. Autophagy (2019) 15:1356–75. doi: 10.1080/15548627.2019.1582743 PMC6628940 30774023

[192] Wu T Jiao L Bai H Hu X Wang M Zhao Z. The dominant model analysis of Sirt3 genetic variants is associated with susceptibility to tuberculosis in a Chinese han population. Mol Genet Genomics (2020) 295:1155–62. doi: 10.1007/s00438-020-01685-7 32462533

[193] Haigis MC Mostoslavsky R Haigis KM Fahie K Christodoulou DC Murphy AJ. SIRT4 inhibits glutamate dehydrogenase and opposes the effects of calorie restriction in pancreatic beta cells. Cell (2006) 126:941–54. doi: 10.1016/j.cell.2006.06.057 16959573

[194] Chang HC Guarente L. SIRT1 and other sirtuins in metabolism. Trends Endocrinol Metab (2014) 25:138–45. doi: 10.1016/j.tem.2013.12.001 PMC3943707 24388149

[195] Tao J Zhang J Ling Y McCall CE Liu TF. Mitochondrial sirtuin 4 resolves immune tolerance in monocytes by rebalancing glycolysis and glucose oxidation homeostasis. Front Immunol (2018) 9:419. doi: 10.3389/fimmu.2018.00419 29593712 PMC5854658

[196] Du J Zhou Y Su X Yu JJ Khan S Jiang H. Sirt5 is a NAD-dependent protein lysine demalonylase and desuccinylase. Science (2011) 334:806–9. doi: 10.1126/science.1207861 PMC3217313 22076378

[197] Nakagawa T Lomb DJ Haigis MC Guarente L. SIRT5 deacetylates carbamoyl phosphate synthetase 1 and regulates the urea cycle. Cell (2009) 137:560–70. doi: 10.1016/j.cell.2009.02.026 PMC2698666 19410549

[198] Lukey MJ Greene KS Cerione RA. Lysine succinylation and SIRT5 couple nutritional status to glutamine catabolism. Mol Cell Oncol (2020) 7:1735284. doi: 10.1080/23723556.2020.1735284 32391426 PMC7199738

[199] Eng CH Yu K Lucas J White E Abraham RT. Ammonia derived from glutaminolysis is a diffusible regulator of autophagy. Sci Signal (2010) 3:ra31. doi: 10.1126/scisignal.2000911 20424262

[200] Polletta L Vernucci E Carnevale I Arcangeli T Rotili D Palmerio S. SIRT5 regulation of ammonia-induced autophagy and mitophagy. Autophagy (2015) 11:253–70. doi: 10.1080/15548627.2015.1009778 PMC4502726 25700560

[201] Rardin MJ He W Nishida Y Newman JC Carrico C Danielson SR. SIRT5 regulates the mitochondrial lysine succinylome and metabolic networks. Cell Metab (2013) 18:920–33. doi: 10.1016/j.cmet.2013.11.013 PMC4105152 24315375

[202] Zhang M Wu J Sun R Tao X Wang X Kang Q. SIRT5 deficiency suppresses mitochondrial ATP production and promotes AMPK activation in response to energy stress. PloS One (2019) 14:e0211796. doi: 10.1371/journal.pone.0211796 30759120 PMC6373945

[203] Zhang Y Bharathi SS Rardin MJ Lu J Maringer KV Sims-Lucas S. Lysine desuccinylase SIRT5 binds to cardiolipin and regulates the electron transport chain. J Biol Chem (2017) 292:10239–49. doi: 10.1074/jbc.M117.785022 PMC5473227 28458255

[204] Gu W Qian Q Xu Y Xu X Zhang L He S. SIRT5 regulates autophagy and apoptosis in gastric cancer cells. J Int Med Res (2021) 49:300060520986355. doi: 10.1177/0300060520986355 33530803 PMC7871096

[205] Qin K Han C Zhang H Li T Li N Cao X. NAD(+) dependent deacetylase sirtuin 5 rescues the innate inflammatory response of endotoxin tolerant macrophages by promoting acetylation of p65. J Autoimmun (2017) 81:120–9. doi: 10.1016/j.jaut.2017.04.006 28461090

[206] McCudden CR Powers-Lee SG. Required allosteric effector site for n-acetylglutamate on carbamoyl-phosphate synthetase I. J Biol Chem (1996) 271:18285–94. doi: 10.1074/jbc.271.30.18285 8663466

[207] Valaei K Mehrabani J Wong A. Effects of l-citrulline supplementation on nitric oxide and antioxidant markers after high-intensity interval exercise in young men: A randomized controlled trial. Br J Nutr (2021), 1–23. 10.1017/S0007114521002178 34134794

[208] Fast W Nikolic D Van Breemen RB Silverman RB. Mechanistic studies of the inactivation of inducible nitric oxide synthase by N^5^-(1-iminoethyl)-L-ornithine (L-NIO). J Am Chrm. Soc (1999) 121:903–16.

[209] Vitecek J Lojek A Valacchi G Kubala L. Arginine-based inhibitors of nitric oxide synthase: therapeutic potential and challenges. Mediators Inflammation (2012) 2012:318087. doi: 10.1155/2012/318087 PMC3441039 22988346

[210] Leiper J Vallance P. Biological significance of endogenous methylarginines that inhibit nitric oxide synthases. Cardiovasc Res (1999) 43:542–8. doi: 10.1016/S0008-6363(99)00162-5 10690326

[211] Vallance P Leone A Calver A Collier J Moncada S. Accumulation of an endogenous inhibitor of nitric oxide synthesis in chronic renal failure. Lancet (1992) 339:572–5. doi: 10.1016/0140-6736(92)90865-Z 1347093

[212] Schrader SM Botella H Jansen R Ehrt S Rhee K Nathan C. Multiform antimicrobial resistance from a metabolic mutation. Sci Adv (2021) 7. doi: 10.1126/sciadv.abh2037 PMC8397267 34452915

[213] Minhas PS Liu L Moon PK Joshi AU Dove C Mhatre S. Macrophage *de novo* NAD(+) synthesis specifies immune function in aging and inflammation. Nat Immunol (2019) 20:50–63. doi: 10.1038/s41590-018-0255-3 30478397 PMC6768398

[214] Cameron AM Castoldi A Sanin DE Flachsmann LJ Field CS Puleston DJ. Inflammatory macrophage dependence on NAD(+) salvage is a consequence of reactive oxygen species-mediated DNA damage. Nat Immunol (2019) 20:420–32. doi: 10.1038/s41590-019-0336-y PMC12842115 30858618

[215] Billingham LK Chandel NS. NAD-biosynthetic pathways regulate innate immunity. Nat Immunol (2019) 20:380–2. doi: 10.1038/s41590-019-0353-x 30858621

[216] Pajuelo D Gonzalez-Juarbe N Tak U Sun J Orihuela CJ Niederweis M. NAD(+) depletion triggers macrophage necroptosis, a cell death pathway exploited by mycobacterium tuberculosis. Cell Rep (2018) 24:429–40. doi: 10.1016/j.celrep.2018.06.042 PMC6136256 29996103

[217] Sun J Siroy A Lokareddy RK Speer A Doornbos KS Cingolani G. The tuberculosis necrotizing toxin kills macrophages by hydrolyzing NAD. Nat Struct Mol Biol (2015) 22:672–8. doi: 10.1038/nsmb.3064 PMC4560639 26237511

[218] Gan H He X Duan L Mirabile-Levens E Kornfeld H Remold HG. Enhancement of antimycobacterial activity of macrophages by stabilization of inner mitochondrial membrane potential. J Infect Dis (2005) 191:1292–300. doi: 10.1086/428906 15776376

[219] Chandra P He L Zimmerman M Yang G Koster S Ouimet M. Inhibition of fatty acid oxidation promotes macrophage control of mycobacterium tuberculosis. mBio (2020) 11. doi: 10.1128/mBio.01139-20 PMC7343992 32636249

[220] Bhaskar A Kumar S Khan MZ Singh A Dwivedi VP Nandicoori VK. Host sirtuin 2 as an immunotherapeutic target against tuberculosis. Elife (2020) 9. doi: 10.7554/eLife.55415 PMC7398663 32697192

[221] Hallows WC Lee S Denu JM. Sirtuins deacetylate and activate mammalian acetyl-CoA synthetases. Proc Natl Acad Sci U.S.A. (2006) 103:10230–5. 10.1073/pnas.0604392103 PMC1480596 16790548

[222] Bosch-Presegue L Vaquero A. Sirtuin-dependent epigenetic regulation in the maintenance of genome integrity. FEBS J (2015) 282:1745–67. doi: 10.1111/febs.13053 25223884

[223] Zhang S Liu Y Zhou X Ou M Xiao G Li F. Sirtuin 7 regulates nitric oxide production and apoptosis to promote mycobacterial clearance in macrophages. Front Immunol (2021) 12:779235. doi: 10.3389/fimmu.2021.779235 34925356 PMC8678072

[224] Smulan L Kornfeld H Singhal A. Sirtuin deacetylases: Linking mycobacterial infection and host metabolism (2021) sirtuin deacetylases: Linking mycobacterial infection and host metabolism. In: Karakousis PC Hafner R Gennaro ML, editors. Advances in host-directed therapies against tuberculosis. Cham: Springer (2021). doi: 10.1007/978-3-030-56905-1_2

[225] Cuyas E Verdura S Llorach-Pares L Fernandez-Arroyo S Joven J Martin-Castillo B. Metformin is a direct SIRT1-activating compound: Computational modeling and experimental validation. Front Endocrinol (Lausanne) (2018) 9:657. doi: 10.3389/fendo.2018.00657 30459716 PMC6232372

[226] Naicker N Sigal A Naidoo K. Metformin as host-directed therapy for TB treatment: Scoping review. Front Microbiol (2020) 11:435. doi: 10.3389/fmicb.2020.00435 32411100 PMC7201016

[227] Kozako T Suzuki T Yoshimitsu M Arima N Honda S Soeda S. Anticancer agents targeted to sirtuins. Molecules (2014) 19:20295–313. doi: 10.3390/molecules191220295 PMC6270850 25486244

[228] Rozwarski DA Grant GA Barton DH Jacobs WR Jr. Sacchettini JC. Modification of the NADH of the isoniazid target (InhA) from mycobacterium tuberculosis. Science (1998) 279:98–102. doi: 10.1126/science.279.5347.98 9417034

[229] Sharma V Grubmeyer C Sacchettini JC. Crystal structure of quinolinic acid phosphoribosyltransferase from mmycobacterium tuberculosis: a potential TB drug target. Structure (1998) 6:1587–99. doi: 10.1016/S0969-2126(98)00156-7 9862811

[230] Kim H Shibayama K Rimbara E Mori S. Biochemical characterization of quinolinic acid phosphoribosyltransferase from mycobacterium tuberculosis H37Rv and inhibition of its activity by pyrazinamide. PloS One (2014) 9:e100062. doi: 10.1371/journal.pone.0100062 24949952 PMC4065032

[231] Malik SS Patterson DN Ncube Z Toth EA. The crystal structure of human quinolinic acid phosphoribosyltransferase in complex with its inhibitor phthalic acid. Proteins (2014) 82:405–14. doi: 10.1002/prot.24406 24038671

[232] Begley TP Kinsland C Mehl RA Osterman A Dorrestein P. The biosynthesis of nicotinamide adenine dinucleotides in bacteria. Vitam Horm (2001) 61:103–19. doi: 10.1016/S0083-6729(01)61003-3 11153263

[233] Jansen RS Mandyoli L Hughes R Wakabayashi S Pinkham JT Selbach B. Aspartate aminotransferase Rv3722c governs aspartate-dependent nitrogen metabolism in mycobacterium tuberculosis. Nat Commun (2020) 11:1960. doi: 10.1038/s41467-020-15876-8 32327655 PMC7181641

[234] Kurita K Ohta H Shirakawa I Tanaka M Kitaura Y Iwasaki Y. Macrophages rely on extracellular serine to suppress aberrant cytokine production. Sci Rep (2021) 11:11137. doi: 10.1038/s41598-021-90086-w 34045514 PMC8160356

[235] Rodriguez AE Ducker GS Billingham LK Martinez CA Mainolfi N Suri V. Serine metabolism supports macrophage IL-1beta production. Cell Metab (2019) 29:1003–1011 e1004. 30773464 10.1016/j.cmet.2019.01.014 PMC6447453

[236] Borah K Beyss M Theorell A Wu H Basu P Mendum TA. Intracellular mycobacterium tuberculosis exploits multiple host nitrogen sources during growth in human macrophages. Cell Rep (2019) 29:3580–3591 e3584. doi: 10.1016/j.celrep.2019.11.037 31825837 PMC6915324

[237] Ji J Xu Y Zheng M Luo C Lei H Qu H. Methionine attenuates lipopolysaccharide-induced inflammatory responses *via* DNA methylation in macrophages. ACS Omega (2019) 4:2331–6. doi: 10.1021/acsomega.8b03571 PMC6374979 30775649

[238] Berney M Berney-Meyer L Wong KW Chen B Chen M Kim J. Essential roles of methionine and s-adenosylmethionine in the autarkic lifestyle of mycobacterium tuberculosis. Proc Natl Acad Sci U.S.A. (2015) 112:10008–13. 10.1073/pnas.1513033112 PMC4538671 26221021

[239] Chaton CT Rodriguez ES Reed RW Li J Kenner CW Korotkov KV. Structural analysis of mycobacterial homoserine transacetylases central to methionine biosynthesis reveals druggable active site. Sci Rep (2019) 9:20267. doi: 10.1038/s41598-019-56722-2 31889085 PMC6937278

[240] Hasenoehrl EJ Rae Sajorda D Berney-Meyer L Johnson S Tufariello JM Fuhrer T. Derailing the aspartate pathway of mycobacterium tuberculosis to eradicate persistent infection. Nat Commun (2019) 10:4215. doi: 10.1038/s41467-019-12224-3 31527595 PMC6746716

[241] Colak G Pougovkina O Dai L Tan M Te Brinke H Huang H. Proteomic and biochemical studies of lysine malonylation suggest its malonic aciduria-associated regulatory role in mitochondrial function and fatty acid oxidation. Mol Cell Proteomics (2015) 14:3056–71. doi: 10.1074/mcp.M115.048850 PMC4638046 26320211

[242] Park J Chen Y Tishkoff DX Peng C Tan M Dai L. SIRT5-mediated lysine desuccinylation impacts diverse metabolic pathways. Mol Cell (2013) 50:919–30. doi: 10.1016/j.molcel.2013.06.001 PMC3769971 23806337

[243] Giblin W Bringman-Rodenbarger L Guo AH Kumar S Monovich AC Mostafa AM. The deacylase SIRT5 supports melanoma viability by influencing chromatin dynamics. J Clin Invest (2021) 131. doi: 10.1172/JCI138926 PMC8203465 33945506

[244] Yang X Wang Z Li X Liu B Liu M Liu L. SHMT2 desuccinylation by SIRT5 drives cancer cell proliferation. Cancer Res (2018) 78:372–86. doi: 10.1158/0008-5472.CAN-17-1912 29180469

[245] Gao X Reid MA Kong M Locasale JW. Metabolic interactions with cancer epigenetics. Mol Aspects Med (2017) 54:50–7. doi: 10.1016/j.mam.2016.09.001 PMC5344790 27620316

[246] Wang C Wan X Yu T Huang Z Shen C Qi Q. Acetylation stabilizes phosphoglycerate dehydrogenase by disrupting the interaction of E3 ligase RNF5 to promote breast tumorigenesis. Cell Rep (2020) 32:108021. doi: 10.1016/j.celrep.2020.108021 32783943

